# HDAC11 promotes both NLRP3/caspase-1/GSDMD and caspase-3/GSDME pathways causing pyroptosis via ERG in vascular endothelial cells

**DOI:** 10.1038/s41420-022-00906-9

**Published:** 2022-03-12

**Authors:** Feng Yao, Zhen Jin, Zihan Zheng, Xiaohan Lv, Lingxuan Ren, Jianjun Yang, Danli Chen, Bo Wang, Wei Yang, Lifang Chen, Weirong Wang, Jianli Gu, Rong Lin

**Affiliations:** 1 Department of Pharmacology, Xi’an Jiaotong University Health Science Center, Xi’an, China; 2 Xi’an NO.3 hospital, Xi’an, China; 3 The First Department of Geriatrics, First Affiliated Hospital of Xi’an Jiaotong University, Xi’an, China; 4 Department of Medical Laboratory Animal Science, Xi’an Jiaotong University Health Science Center, Xi’an, China

**Keywords:** Atherosclerosis, Mechanisms of disease

## Abstract

Histone deacetylase 11 (HDAC11), a sole member of the class IV HDAC subfamily, participates in various cardiovascular diseases. Recent evidence showed that pyroptosis was a form of inflammatory programmed cell death and is critical for atherosclerosis (AS). However, little is known about the effect of HDAC11 on endothelial cell pyroptosis in AS. Thus, this study aims to investigate the role of HDAC11 in vascular endothelial cell pyroptosis and its molecular mechanism. Firstly, we found that HDAC11 expression was up-regulated and pyroptosis occurred in the aorta of ApoE^−/−^ mice fed with a high-fat diet (HFD) for 8 or 12 weeks. Then, in vitro study found the treatment of human umbilical vein endothelial cells (HUVECs) with tumor necrosis factor-α (TNF-α) resulted in pyroptosis, as evidenced by activation of caspase-1 and caspase-3 activation, cleavage of downstream gasdermin D (GSDMD) and gasdermin E (GSDME/DFNA5), the release of pro-inflammatory cytokines interleukin (IL)-1β, IL-6 and IL-18, as well as elevation of LDH activity and increase of propidium iodide (PI)-positive cells. Besides, TNF-α increased HDAC11 expression and induced pyroptosis via TNFR1 in HUVECs. HDAC11 knockdown mitigated pyroptosis by suppressing both NLRP3/caspase-1/GSDMD and caspase-3/GSDME pathways in TNF-α-induced HUVECs. Moreover, GSDME knockdown by siRNA significantly decreased pyroptosis and inflammatory response, while treatment with disulfiram or necrosulfonamide (NSA) further augmented the inhibitory effects of GSDME siRNA on pyroptosis and inflammatory response. Further studies found HDAC11 formed a complex with ERG and decreased the acetylation levels of ERG. More importantly, ERG knockdown augmented vascular endothelial cell pyroptosis in TNF-α-induced HUVECs. Taken together, our study suggests that HDAC11 might promote both NLRP3/caspase-1/GSDMD and caspase-3/GSDME pathways leading to pyroptosis via regulation of ERG acetylation in HUVECs. Modulation of HDAC11 may serve as a potential target for therapeutic strategies of AS.

## Introduction

Histone deacetylase 11 (HDAC11), the only class IV HDAC member, is reported to regulate immune activation [1], tumorigenesis [2], and cardiovascular diseases [3–5]. It has been found that HDAC11 protein expression was significantly elevated both in human atherosclerotic tissue samples and in the atherosclerotic aorta of ApoE^−/^^−^ mice fed with a high-fat diet (HFD) for 14 weeks [6]. Therefore, HDAC11 may participate in the atherosclerosis (AS) process. In addition, HDAC11 deletion reduced tumor necrosis factor-α (TNF-α) and interleukin (IL)-1β levels in serum and heart tissues of mice fed with fructose [3]. It has been reported that reconstitution of HDAC11 reduced IL-13 expression, while inhibition of HDAC11 increased IL-13 expression in CD4+ T cells of heart tissue in patients with myocarditis [7]. Another study found that inhibition of HDAC11 restored the IL-10 expression in B cells from allergic rhinitis patients [8]. These studies indicated that HDAC11 participated in the regulation of inflammation. Pyroptosis is a pro-inflammatory form of programmed cell death, which is accompanied by the release of a large number of pro-inflammatory cytokines and plays a key role in the initiation and progression of AS [9, 10]. However, whether HDAC11 participates in the regulation of pyroptosis in AS process remains unknown.

Pyroptosis is characterized predominantly by the formation of gasdermin protein family-mediated membrane perforation and the consecutive release of pro-inflammatory cytokines [9–12]. Canonical and non-canonical inflammasome activation of caspase-1/11 (mouse) or caspase-1/4/5 (human) leads to gasdermin D (GSDMD) cleavage and maturation of IL-1β and IL-18, and then the GSDMD-N domain causes pyroptosis by promoting the formation of membrane pores and the release of pro-inflammatory cytokines [11, 12]. It has been found that nicotine promotes AS via NLRP3/caspase-1-mediated endothelial cell pyroptosis in HFD-fed ApoE^−/−^ mice [13]. Another study showed that trimethylamine N-oxide promotes ApoE^−/−^ mice AS by inducing vascular endothelial cell pyroptosis via the NLRP3/caspase-1/GSDMD pathway [14]. In addition, gasdermin E (GSDME/DFNA5) was also found to be expressed in the artery [15]. GSDME cleavage by caspase-3 liberates the GSDME-N domain, which also mediates pyroptosis by forming pores membrane pores [16]. It has been shown that HDAC11 deletion reduced cleavage of caspase-3 in the heart of fructose-fed mice [3]. Therefore, it is necessary to investigate whether HDAC11 is involved in endothelial cell pyroptosis through NLRP3/caspase-1/GSDMD or caspase-3/GSDME pathway in AS process. In addition, it has been unraveled that HDAC11 formed a complex with the ETS-related gene (ERG) to attenuate the ERG gene transcription activities and compromise the vascular endothelial barrier function [5]. ERG is a member of the ETS transcription factor family, has emerged as a major regulator of endothelial function, such as endothelial homeostasis, vascular development, and angiogenesis [17–19]. It has been reported that loss of ERG expression was associated with AS [18]. Moreover, ERG inhibits vascular inflammation by repressing NF-κB activation and pro-inflammatory gene expression in endothelial cells [20]. However, whether the regulation of HDAC11 on endothelial cell pyroptosis is related to ERG remains unknown.

Therefore, in this study, we observed HDAC11 expression in aortic intima of ApoE^−/−^ mice at different stages of AS, and then investigated whether the effect of HDAC11 on endothelial cell pyroptosis was mediated by NLRP3/caspase-1/GSDMD or caspase-3/GSDME pathway in TNF-α-induced human umbilical vein endothelial cells (HUVECs). Furthermore, we investigated whether the regulation of HDAC11 on endothelial cell pyroptosis was related to ERG.

## Materials and methods

### Animal experiments

Eight-week-old male ApoE^−/−^ mice in the C57/BL6 background were obtained from The Jackson Laboratory (Bar Harbor, ME, USA). The experimental protocol was following the National Institutes of Health Guide for the Care and Use of Laboratory Animals and was approved by the Animal Care and Use Committee of Xi’an Jiaotong University. Mice were randomized into four groups (*n* = 10) and were fed an HFD for 0, 4, 8, or 12 weeks. Then all mice were fasted overnight and sacrificed. The aortic root tissues along with the basal portion of the heart of four mice were fixed with 4% paraformaldehyde solution, embedded in optimum cutting temperature compound (SAKURA, USA), and then cut cross-sectionally into sections (7 μm). Sections of three mice were stained with hematoxylin and eosin (H&E) and oil red O (Solarbio, Beijing, China). Images were captured using a fluorescence microscope (Olympus TL4, Japan). The atherosclerotic lesion area of aortic root sections was analyzed by Image-Pro Plus. Each mouse was selected for any experiment by the researchers who did not participate in the study.

### Cell culture and treatment

HUVECs (CRL-1730 cells) were purchased from the American Type Culture Collection (ATCC, Manassas, USA). In brief, HUVECs were grown in DMEM/F-12 medium supplemented with 10% fetal bovine serum (Gibco, Grand Island, New York, USA) and 100 U/ml penicillin–streptomycin in a humidified atmosphere. When the cells were grown to confluence, the culture medium was replaced with a serum-free medium for 24 h incubation before experimental use. Human HDAC11 small interfering RNA (siRNA), GSDME siRNA, ERG siRNA, and negative control (NC) siRNA were chemically synthesized by Shanghai GenePharma Corporation (SGC, China). The siRNA and lipofectamine 2000 (Invitrogen, Carlsbad, California, USA) were separately diluted in serum-free DMEM/F-12 and incubated for 5 min. Then the two solutions were softly mixed and incubated for 20 min, followed by addition to the cells. After 48 h, cells were pretreated with disulfiram or NSA (MedchemExpress, New Jersey, USA) for 1 h and then incubated with TNF-α for 12 h. Disulfiram and NSA were dissolved in DMSO.

### Immunofluorescence

The sections of the aortic root were prepared and incubated with rabbit anti-HDAC11 (1:100, Abcam, ab18973) and mouse anti-CD31 (1:100, Proteintech, Wuhan, China, 66065-2-Ig), overnight at 4 °C. The HUVECs were fixed with 4% paraformaldehyde solution for 30 min, permeabilized with 0.3% Triton X-100 for 30 min, and then blocked with normal goat serum for 1 h at room temperature. Subsequently, the samples were incubated with anti-HDAC11 antibody (1:100, Abcam, ab18973) or anti-ERG antibody (1:50, Proteintech, 14356-1-AP) overnight at 4 °C, followed by incubation with goat anti-rabbit IgG (H&L) - Alexa Fluor 594 (1:200, ImmunoWay Biotechnology Company, Plano, Texas, USA, RS3611) and goat anti-mouse IgG (H&L) - Alexa Fluor 488 (1:200, ImmunoWay Biotechnology Company, RS3208) for 2 h at room temperature. Nuclei were stained with DAPI (Beyotime, Haimen, China). The samples were observed under a fluorescence microscope.

### Cytokine analysis by ELISA

All blood samples were obtained by penetrating the retro-orbital sinus in mice, and the serum was separated by centrifugation at 3000 rpm for 15 min at 4 °C. TNF-α levels in serum and IL-6 levels in cellular supernatant of HUVECs were detected by ELISA assay kit (TBhealthcare, Foshan, China), IL-1β and IL-18 levels in the serum and cellular supernatant were detected by ELISA assay kit (Ruixin Biotech, Quanzhou, China) according to the manufacturer’s instructions.

### Cytotoxicity assay

Cytotoxicity was determined by measuring LDH released from HUVECs using the cytotoxicity detection LDH kit (Beyotime, Haimen, China) according to the manufacturer’s instructions. The absorbance was determined at a wavelength of 450 nm using a microplate reader (Thermo Fisher, USA).

### Hoechst 33342/PI fluorescent staining

To assess pyroptosis, cells were double-stained with Hoechst 33342 and PI. HUVECs were seeded in 12-well plates in complete medium and treated with TNF-α, disulfiram or NSA. After the indicated treatments, the cells in each group were stained with Hoechst 33342 staining solution for live cells and 2 µg/mL PI (Solarbio, Beijing, China) for 20 min. Then the cells were washed with PBS three times. The cells were photographed under a fluorescence microscope.

### Caspase-1 and Caspase-3 activity analysis

HUVECs were seeded in 6-well plates in complete medium and treated with TNF-α or siRNA. After the indicated treatments, cells were collected. Then the caspase-1 or caspase-3 activity was assayed using the Caspase-1 or Caspase-3 activity assay kit (Beyotime, Haimen, China) according to the manufacturer’s instructions.

### Western blotting

Protein was extracted from the thoracic aorta of mice and HUVECs with RIPA lysis buffer containing protease inhibitor cocktail (Roche, Mannheim, Germany). Protein samples were separated by sodium dodecyl sulfate-polyacrylamide gel electrophoresis and transferred to polyvinylidene difluoride membranes. The membranes were blocked with five percent skimmed milk at room temperature for 2–3 h and then incubated overnight at 4 °C with antibodies. The antibodies used were HDAC11 (1:250, Abcam, ab18973), GSDMD (1:1000, Cell Signaling Technology, #93709), GSDMD-N (1:1000, Cell Signaling Technology, #36425), GSDME (1:500, Proteintech, 13075-1-AP), GSDME-N (1:1000, Abcam, ab222407 and ab222408), TNFR1 (1:500, Wanleibio, Shenyang, China, WL01414), TNFR2 (1:500, Wanleibio, WL02956), pro-caspase-1 (1:1000, Wanleibio, WL02996), cleaved caspase-1 (1:1000, Wanleibio, WL02996a), NLRP3 (1:1000, Wanleibio, WL02635), ASC (1:1000, ImmunoWay Biotechnology Company, YT0365), pro-caspase-3 (1:500, Wanleibio, WL04004), cleaved caspase-3 (1:500, Wanleibio, WL01992), ERG (1:500, Proteintech, 14356-1-AP), and mouse anti-β-actin (1:1000, Cell Signaling Technology, #58169S). Secondary antibody and the enhanced chemiluminescent substrate were used for detection. Relative protein expression levels were semiquantitatively performed using Lane 1D software (Sage Creation Science Co, China).

### RNA extraction and quantitative real-time PCR

TRIzol reagent (Invitrogen, Carlsbad, California, USA) was applied to extract total RNA from the thoracic aorta of mice and HUVECs. All-in-One cDNA Synthesis SuperMix (Bimake, Houston, Texas, USA) was used for the reverse transcription of the RNA into cDNA. Quantitative real-time PCR was performed with 2× SYBR Green qPCR Master Mix by StepOnePlus^™^ Real-Time PCR System (Thermo Fisher, Massachusetts, USA). Relative changes in mRNA levels were analyzed by the ^ΔΔ^CT method. The sequences of primer pairs are shown in Table S1.

### Immunoprecipitation

HUVECs were seeded in 6-well plates in complete medium and treated with TNF-α or siRNA. Cells were washed with PBS and collected, the protein lysates were incubated with acetylated-lysine, ERG or IgG antibody and precipitated with Protein A/G Magnetic Beads (Bimake, Houston, Texas, USA) at 4 °C. Protein A/G Magnetic Beads were collected, washed with lysis buffer four times, and then the target protein was detected by Western blotting.

### Statistical analysis

All quantitative data are presented as the mean ± SD from three or more independent experiments. Statistical analysis was undertaken using GraphPad Prism (GraphPad Software, La Jolla, CA, USA). Multiple comparisons were assessed by one-way analysis of variance (ANOVA). A *P* value < 0.05 was considered statistically significant.

## Results

### HDAC11 expression is upregulated and pyroptosis is activated in the aorta of HFD-fed ApoE^−/−^ mice

**Fig. 1:**
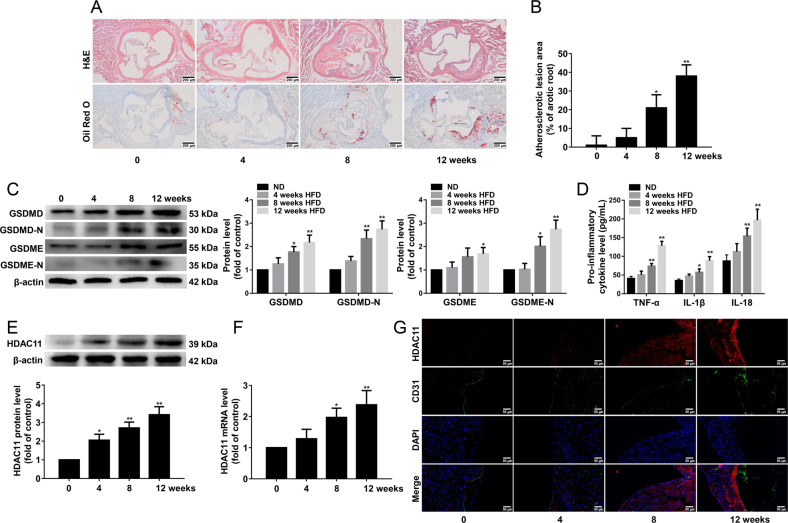
HDAC11 expression is upregulated and pyroptosis is activated in the aorta of HFD-fed ApoE^−/−^ mice. ApoE^−/−^ mice were fed an HFD for 0, 4, 8, or 12 weeks. **A** Representative images showing the atherosclerotic lesions of aortic root sections with H&E staining and the lipid deposition of aortic root sections with Oil red O staining, ×100. Scale bar indicates 200 μm. **B** The atherosclerotic lesion area of aortic root sections was analyzed (*n* = 3). **C** GSDMD, GSDMD-N, GSDME, and GSDME-N protein expressions in the aorta were determined by Western blotting (*n* = 3). **D** The levels of TNF-α, IL-1β, and IL-18 in the serum were determined by ELISA (*n* = 10). **E**, **F** HDAC11 protein and mRNA expressions in the aorta were determined by Western blotting and quantitative real-time PCR (*n* = 3). **G** The expression of HDAC11 in aortic intima by immunofluorescent double staining of the aortic sinus of HDAC11 and CD31. The nuclei were stained blue with DAPI. Scale bar indicates 50 μm. *P < 0.05, **P < 0.01 vs. ND group. Results are expressed as mean ± SD.

Our results showed that AS was successfully induced in ApoE^−/−^ mice after HFD feeding for 8 weeks (Fig. 1A, B). GSDMD-N and GSDME-N play an important role in pyroptosis. GSDMD, GSDMD-N, and GSDME-N protein expressions were significantly increased in the aorta of ApoE^−/−^ mice after HFD feeding for 8 weeks (Fig. 1C). Moreover, the serum levels of TNF-α, IL-1β, and IL-18 were significantly increased in ApoE^−/−^ mice after HFD feeding for 8 weeks (Fig. 1D). Our findings suggested that HFD results in pyroptosis in the aorta of ApoE^−/−^ mice. To examine whether HDAC11 is related to vascular pyroptosis of AS process, we determined the HDAC11 expression in the aorta of ApoE^−/−^ mice. We found that HDAC11 protein and mRNA expressions were significantly increased in the aorta of ApoE^−/−^ mice after HFD feeding for 8 weeks (Fig. 1E, F). Furthermore, HDAC11 protein expression was increased in aortic intima of HFD-fed ApoE^−/−^ mice by immunofluorescent double staining of the aortic sinus of HDAC11 and CD31 (Fig. 1G).

### TNF-α induces pyroptosis and upregulates HDAC11 expression via TNFR1 in HUVECs

**Fig. 2:**
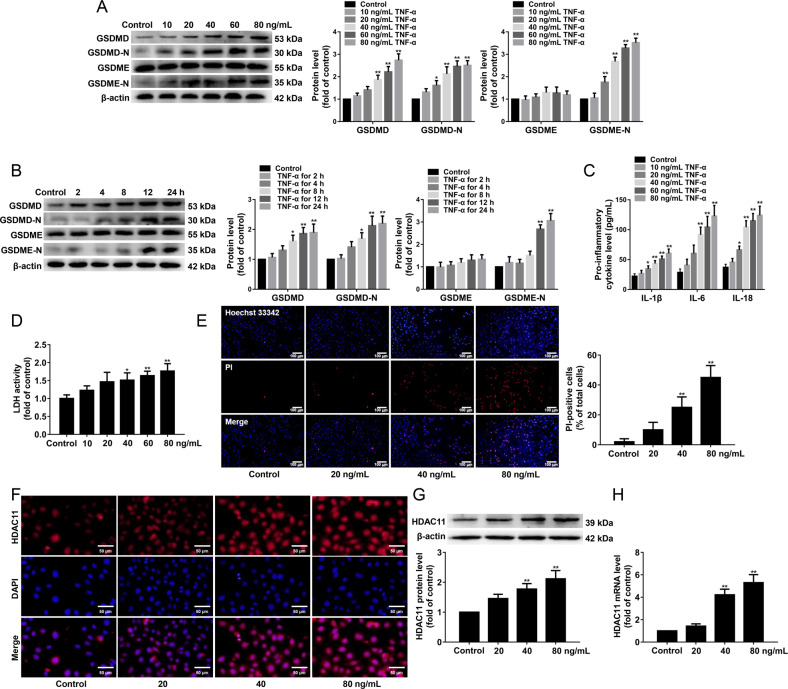
TNF-α induces pyroptosis and upregulates HDAC11 expression in HUVECs. **A** HUVECs were treated with TNF-α (10, 20, 40, 60, or 80 ng/mL) for 12 h. GSDMD, GSDMD-N, GSDME, and GSDME-N protein expressions were determined by Western blotting. **B** HUVECs were treated with 40 ng/mL TNF-α for 2, 4, 8, 12, or 24 h. GSDMD, GSDMD-N, GSDME, and GSDME-N protein expressions were determined by Western blotting. **C** The levels of IL-1β, IL-6, and IL-18 in the cellular supernatant were detected by ELISA assay. **D** The cellular supernatant LDH level was evaluated with a cytotoxicity detection LDH kit. **E** The representative photographs of double-fluorescent staining with PI (red) and Hoechst 33342 (blue), and the quantification of PI-positive cells, ×200. Scale bar indicates 100 μm. **F** Immunofluorescence staining was performed to determine the expression and localization of HDAC11, ×200. Scale bar indicates 50 μm. **G**, **H** The protein and mRNA expressions of HDAC11 were determined by Western blotting and quantitative real-time PCR. *P < 0.05, **P < 0.01 vs. control group. Results are expressed as mean ± SD (*n* = 3).

To investigate whether TNF-α induces endothelial cell pyroptosis, HUVECs were treated with 10, 20, 40, 60, or 80 ng/mL TNF-α for 12 h or 40 ng/mL TNF-α for 2, 4, 8, 12, or 24 h, and the protein expressions of GSDMD, GSDMD-N, GSDME, and GSDME-N were detected. We found that TNF-α had no effects on GSDME protein expression, while GSDMD, GSDMD-N, and GSDME-N protein expressions were significantly increased in HUVECs treated with 40, 60, or 80 ng/mL TNF-α for 12 h or 40 ng/mL TNF-α for 12 or 24 h compared with the control group (Fig. 2A, B). In addition, we found that 40, 60, and 80 ng/mL TNF-α significantly increased the release of pro-inflammatory cytokines such as IL-1β, IL-6, and IL-18 (Fig. 2C). Moreover, we further found that 40 ng/mL TNF-α significantly increased the cellular supernatant LDH activity (Fig. 2D) and the number of PI-positive cells (Fig. 2E). To investigate whether TNF-α affects the subcellular localization and expression of HDAC11, we performed immunofluorescence staining. We found that TNF-α treatment significantly elevated HDAC11 protein expression, did not alter HDAC11 subcellular localization (Fig. 2F). In addition, TNF-α significantly increased HDAC11 protein and mRNA expressions by Western blotting and quantitative real-time PCR (Fig. 2G, H).

**Fig. 3:**
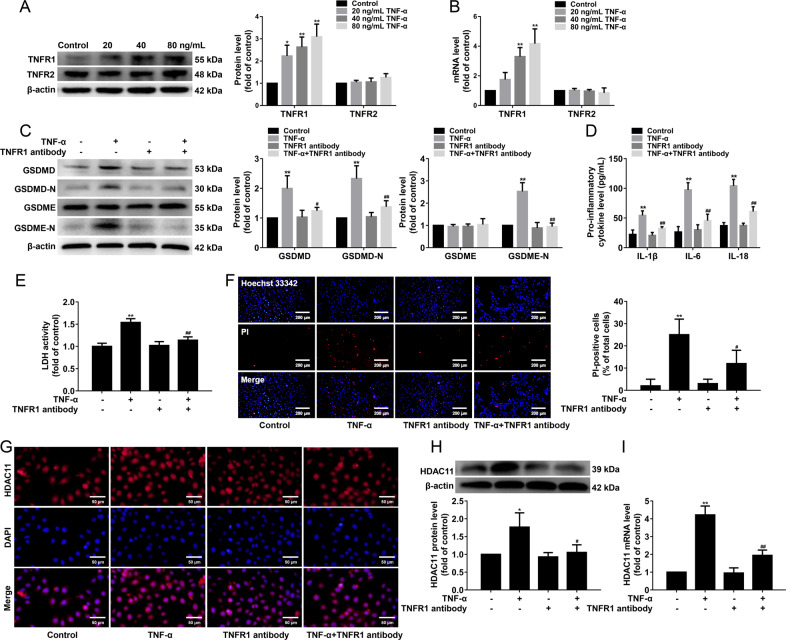
Blockade of TNFR1 inhibits pyroptosis and downregulates HDAC11 expression in TNF-α-induced HUVECs. HUVECs were treated with TNF-α (20, 40, or 80 ng/mL) for 12 h. **A**, **B** The expressions of TNFR1 and TNFR2 protein and mRNA were determined by Western blotting and quantitative real-time PCR, respectively. The HUVECs were pretreated with a TNFR1 neutralizing antibody (5 µg/mL) for 30 min and then stimulated with TNF-α (40 ng/mL) for 12 h. **C** The protein expressions of GSDMD, GSDMD-N, GSDME, and GSDME-N were determined by Western blotting. **D** The levels of IL-1β, IL-6, and IL-18 in the cellular supernatant were detected by ELISA assay. **E** The cellular supernatant LDH level was evaluated with a cytotoxicity detection LDH kit. **F** The representative photographs of double-fluorescent staining with PI (red) and Hoechst 33342 (blue), and the quantification of PI-positive cells, ×100. Scale bar indicates 200 μm. **G** Immunofluorescence staining was performed to determine the expression and localization of HDAC11, ×200. Scale bar indicates 50 μm. **H**, **I** The protein and mRNA expressions of HDAC11 were determined by Western blotting and quantitative real-time PCR. **P < 0.01, vs. NC siRNA group; ^#^*P* < 0.05, ^##^*P* < 0.01 vs. NC siRNA ^+^ TNF-α group. Results are expressed as mean ± SD (*n* = 3).

We found that TNF-α significantly increased TNFR1 protein and mRNA expressions, while TNF-α had no effects on TNFR2 protein and mRNA expressions compared with the control group in HUVECs (Fig. 3A, B). Therefore, we explored whether TNF-α induced pyroptosis via TNFR1. The HUVECs were pretreated with TNFR1 neutralizing antibody (5 µg/mL) for 30 min and then stimulated with 40 ng/mL TNF-α for 12 h. Our results showed that blockade of TNFR1 using neutralizing antibody inhibited the protein expressions of GSDMD-N and GSDME-N in TNF-α-induced HUVECs (Fig. 3C). In addition, the blockade of TNFR1 significantly suppressed the release of IL-1β, IL-6, and IL-18 (Fig. 3D). Further study found that blockade of TNFR1 significantly reduced the cellular supernatant LDH activity (Fig. 3E) and the number of PI-positive cells (Fig. 3F). These results suggested that TNF-α induced pyroptosis via TNFR1 in HUVECs. Moreover, we further explored whether TNF-α upregulated HDAC11 expression via TNFR1. Our results showed that blockade of TNFR1 inhibited HDAC11 protein and mRNA expressions in TNF-α-induced HUVECs (Fig. 3G, H and I). Our findings suggested that TNF-α induced pyroptosis and upregulated HDAC11 expression via TNFR1 in HUVECs.

### HDAC11 knockdown inhibits pyroptosis in TNF-α-induced HUVECs

**Fig. 4:**
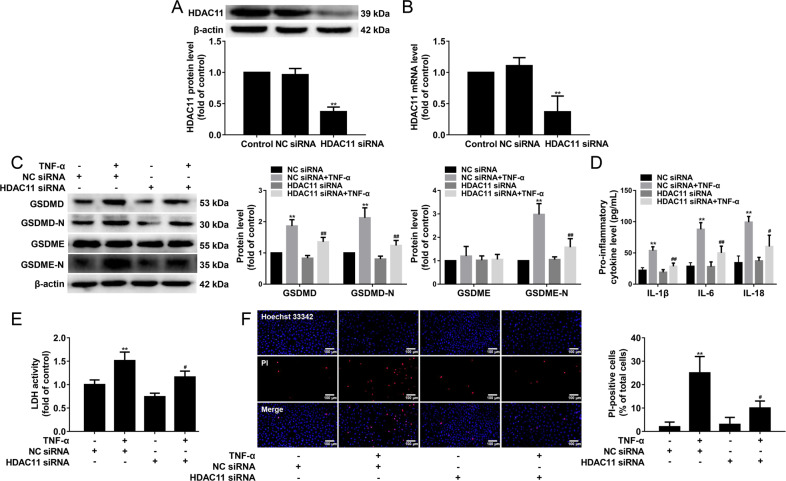
HDAC11 knockdown inhibits pyroptosis in TNF-α-induced HUVECs. **A**, **B** HUVECs were transfected with HDAC11 siRNA or NC siRNA for 48 h, and then the protein and mRNA expressions of HDAC11 were determined by western blotting and quantitative real-time PCR. HUVECs were stimulated with 40 ng/mL TNF-α for 12 h after their transfection with HDAC11 siRNA or NC siRNA. **C** The protein expressions of GSDMD, GSDMD-N, GSDME, and GSDME-N were determined by Western blotting. **D** The levels of IL-1β, IL-6, and IL-18 in the cellular supernatant were detected by ELISA assay. **E** The cellular supernatant LDH level was evaluated with a cytotoxicity detection LDH kit. **F** The representative photographs of double-fluorescent staining with PI (red) and Hoechst 33342 (blue), and the quantification of PI-positive cells, ×200. Scale bar indicates 100 μm. **P < 0.01, vs. NC siRNA group; ^#^*P* < 0.05, ^##^*P* < 0.01 vs. NC siRNA ^+^ TNF-α group. Results are expressed as mean ± SD (*n* = 3).

To assess the role of HDAC11 in TNF-α-induced endothelial cell pyroptosis, HUVECs were transfected with HDAC11 siRNA or NC siRNA for 48 h, and then the transfection efficiency of HDAC11 into the HUVECs was detected by Western blotting and quantitative real-time PCR, respectively. The results showed that the efficiency of HDAC11 silencing in HUVECs was approximately 60% at protein and mRNA levels (Fig. 4A, B). We then evaluated the effect of HDAC11 knockdown by siRNA on pyroptosis in HUVECs treated with 40 ng/mL TNF-α for 12 h. HDAC11 knockdown significantly decreased the protein expressions of GSDMD-N and GSDME-N in TNF-α-induced HUVECs (Fig. 4C). In addition, HDAC11 knockdown significantly suppressed the release of IL-1β, IL-6, and IL-18 in TNF-α-induced HUVECs (Fig. 4D). Further study found that HDAC11 knockdown significantly reduced the cellular supernatant LDH activity (Fig. 4E) and the number of PI-positive cells in TNF-α-induced HUVECs (Fig. 4F). These results suggest that HDAC11 knockdown could partly protect HUVECs from TNF-α-induced endothelial cell pyroptosis.

### HDAC11 knockdown suppresses both NLRP3/caspase-1/GSDMD and caspase-3/GSDME pathways in TNF-α-induced HUVECs

**Fig. 5:**
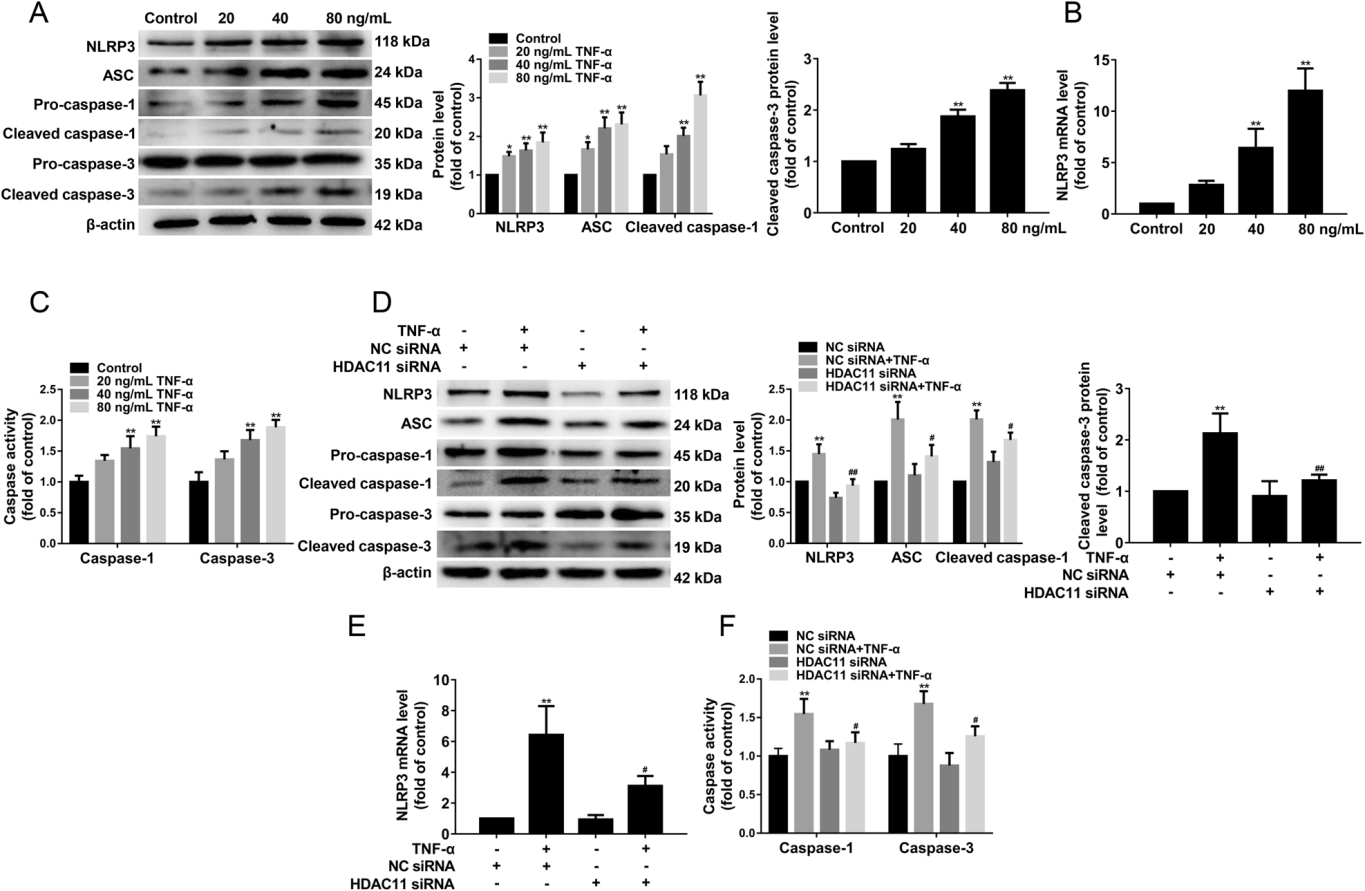
HDAC11 knockdown suppresses both NLRP3/caspase-1/GSDMD and caspase-3/GSDME pathways in TNF-α-induced HUVECs. HUVECs were treated with TNF-α (20, 40, or 80 ng/mL) for 12 h. **A** The expressions of NLRP3, ASC, pro-caspase-1, cleaved caspase-1, pro-caspase-3, and cleaved caspase-3 were determined by Western blotting. **B** NLRP3 mRNA expression was assayed by quantitative real-time PCR. **C** Caspase-1 and caspase-3 activity were determined using the Caspase-1 activity assay kit and the Caspase-3 activity assay kit. *P < 0.05, **P < 0.01 vs. control group. **D** HUVECs were stimulated with 40 ng/mL TNF-α for 12 h after their transfection with HDAC11 siRNA or NC siRNA for 48 h. The expressions of NLRP3, ASC, pro-caspase-1, cleaved caspase-1, pro-caspase-3, and cleaved caspase-3 were determined by Western blotting. **E** NLRP3 mRNA expression was assayed by quantitative real-time PCR. **F** Caspase-1 and caspase**-**3 activity were determined using the Caspase-1 activity assay kit and the Caspase-3 activity assay kit. **P < 0.01 vs. NC siRNA group; ^#^*P* < 0.05, ^##^*P* < 0.01 vs. NC siRNA + TNF-α group. Results are expressed as mean ± SD (*n* = 3).

We found that NLRP3, ASC, cleaved caspase-1, cleaved caspase-3 protein expressions were significantly increased in the aorta of ApoE^−/−^ mice after HFD feeding for 12 weeks (Fig. S1A). As shown in Fig. S1B, cleaved caspase-1 protein expression in aortic intima of HFD-fed ApoE^−/−^ mice was increased by immunofluorescent double staining of the aortic sinus of cleaved caspase-1 and CD31. Moreover, we found that TNF-α significantly increased NLRP3, ASC, cleaved caspase-1 and cleaved caspase-3 protein expressions (Fig. 5A), NLRP3 mRNA expression (Fig. 5B), and caspase-1 and caspase-3 activities (Fig. 5C). HDAC11 knockdown significantly suppressed cleavage of GSDMD and GSDME (Fig. 4C). Therefore, we further examine the effect of HDAC11 knockdown on NLRP3/caspase-1/GSDMD and caspase-3/GSDME pathways. Our results showed that HDAC11 knockdown resulted in a significant decrease in the protein expressions of NLRP3, ASC, cleaved caspase-1, and cleaved caspase-3 (Fig. 5D). Additionally, HDAC11 knockdown significantly decreased NLRP3 mRNA expression (Fig. 5E), and caspase-1 and caspase-3 activities (Fig. 5F). Together, these results confirmed that HDAC11 knockdown inhibited both NLRP3/caspase-1/GSDMD and caspase-3/GSDME pathways in TNF-α-induced HUVECs.

### GSDMD inhibitors and GSDME knockdown suppress pyroptosis and inflammatory response in TNF-α-induced HUVECs

**Fig. 6:**
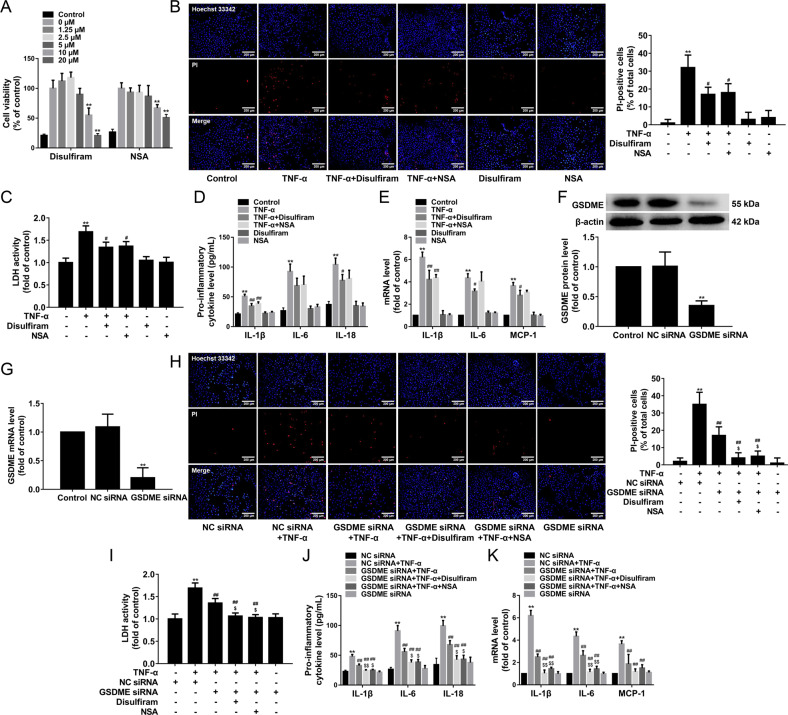
GSDMD inhibitors and GSDME knockdown inhibit pyroptosis and inflammatory response in TNF-α-induced HUVECs. **A** HUVECs were treated with different concentrations of disulfiram and NSA (1.25, 2.5, 5, 10, or 20 μmol/L) for 13 h.The cell viability was evaluated by MTT assay (*n* = 6). **B** HUVECs were pretreated with disulfiram or NSA (5 μmol/L) for 1 h and then stimulated with TNF-α for 12 h. The representative photographs of double-fluorescent staining with PI (red) and Hoechst 33342 (blue), and the quantification of PI-positive cells, ×100. Scale bar indicates 200 μm. **C** The cellular supernatant LDH level was evaluated with a cytotoxicity detection LDH kit. **D** The levels of IL-1β, IL-6, and IL-18 in the cellular supernatant were detected by ELISA assay. **E** The mRNA expressions of IL-1β, IL-6, and MCP-1 were determined by quantitative real-time PCR. **F**, **G** HUVECs were transfected with GSDME siRNA or NC siRNA for 48 h, and then the protein and mRNA expressions of GSDME were determined by western blotting and quantitative real-time PCR. **H** HUVECs were pretreated with disulfiram or NSA for 1 h and then incubated with TNF-α for 12 h after their transfection with GSDME siRNA or NC siRNA. The representative photographs of double-fluorescent staining with PI (red) and Hoechst 33342 (blue), and the quantification of PI-positive cells, ×100. Scale bar indicates 200 μm. **I** The cellular supernatant LDH level was evaluated with a cytotoxicity detection LDH kit. **J** The levels of IL-1β, IL-6, and IL-18 in the cellular supernatant were detected by ELISA assay. **K** The mRNA expressions of IL-1β, IL-6, and MCP-1 were determined by quantitative real-time PCR. **P < 0.01 vs. control group or NC siRNA group; ^#^*P* < 0.05, ^##^*P* < 0.01 vs. TNF-α group or NC siRNA + TNF-α group; ^$^*P* < 0.05, ^$$^*P* < 0.01 vs. GSDME siRNA + TNF-α group. Results are expressed as mean ± SD (*n* = 3).

Pyroptosis inhibitors disulfiram and NSA inhibited pyroptosis by blocking GSDMD pore formation. To determine the toxic effects of disulfiram and NSA, the HUVECs were treated with different concentrations of disulfiram and NSA (1.25, 2.5, 5, 10, or 20 μmol/L) for 13 h. The cell viability was evaluated by MTT assay. Our results found that 1.25, 2.5, and 5 μmol/L disulfiram and NSA had no effects on the cell viability, while 10 and 20 μmol/L disulfiram and NSA significantly decreased the cell viability (Fig. 6A). To further investigate the effects of disulfiram and NSA on TNF-α-induced endothelial cell pyroptosis, the HUVECs were pretreated with disulfiram or NSA (5 μmol/L) for 1 h and then stimulated with TNF-α (40 ng/mL) for 12 h. We found that disulfiram and NSA significantly decreased TNF-α-induced pore formation and membrane rupture by decreasing the number of PI-positive cells in TNF-α-induced HUVECs (Fig. 6B) and the cellular supernatant LDH activity (Fig. 6C). Moreover, disulfiram significantly suppressed the release of IL-1β and IL-18, NSA significantly suppressed the release of IL-1β (Fig. 6D). We further found that disulfiram significantly decreased IL-1β, IL-6, and MCP-1 mRNA expressions, NSA significantly decreased IL-1β mRNA expression compared with the TNF-α group (Fig. 6E).

To further examine whether the effect of GSDMD inhibitors and GSDME knockdown on pyroptosis and inflammatory response, HUVECs were transfected with GSDME siRNA or NC siRNA for 48 h, and then cells were pretreated with disulfiram or NSA for 1 h and exposed to 40 ng/mL TNF-α for an additional 12 h. The results showed that the efficiency of GSDME silencing in HUVECs was approximately 65% at the protein level and 80% at the mRNA level (Fig. 6F, G). GSDME knockdown significantly decreased TNF-α-induced pore formation and membrane rupture by decreasing the number of PI-positive cells and the cellular supernatant LDH activity, while disulfiram and NSA further suppressed the number of PI-positive cells and the cellular supernatant LDH activity (Fig. 6H, I). Moreover, GSDME knockdown significantly decreased inflammatory response by suppressing the release of IL-1β, IL-6, and IL-18, decreasing IL-1β, IL-6, and MCP-1 mRNA expressions, while treatment with disulfiram and NSA further augmented the inhibitory effects of GSDME siRNA on the inflammatory response (Fig. 6J, K). Together, our results confirmed that HDAC11 regulated GSDMD- and GSDME-mediated pyroptosis in TNF-α-induced HUVECs.

### HDAC11 regulates pyroptosis via ERG in TNF-α-induced HUVECs

**Fig. 7:**
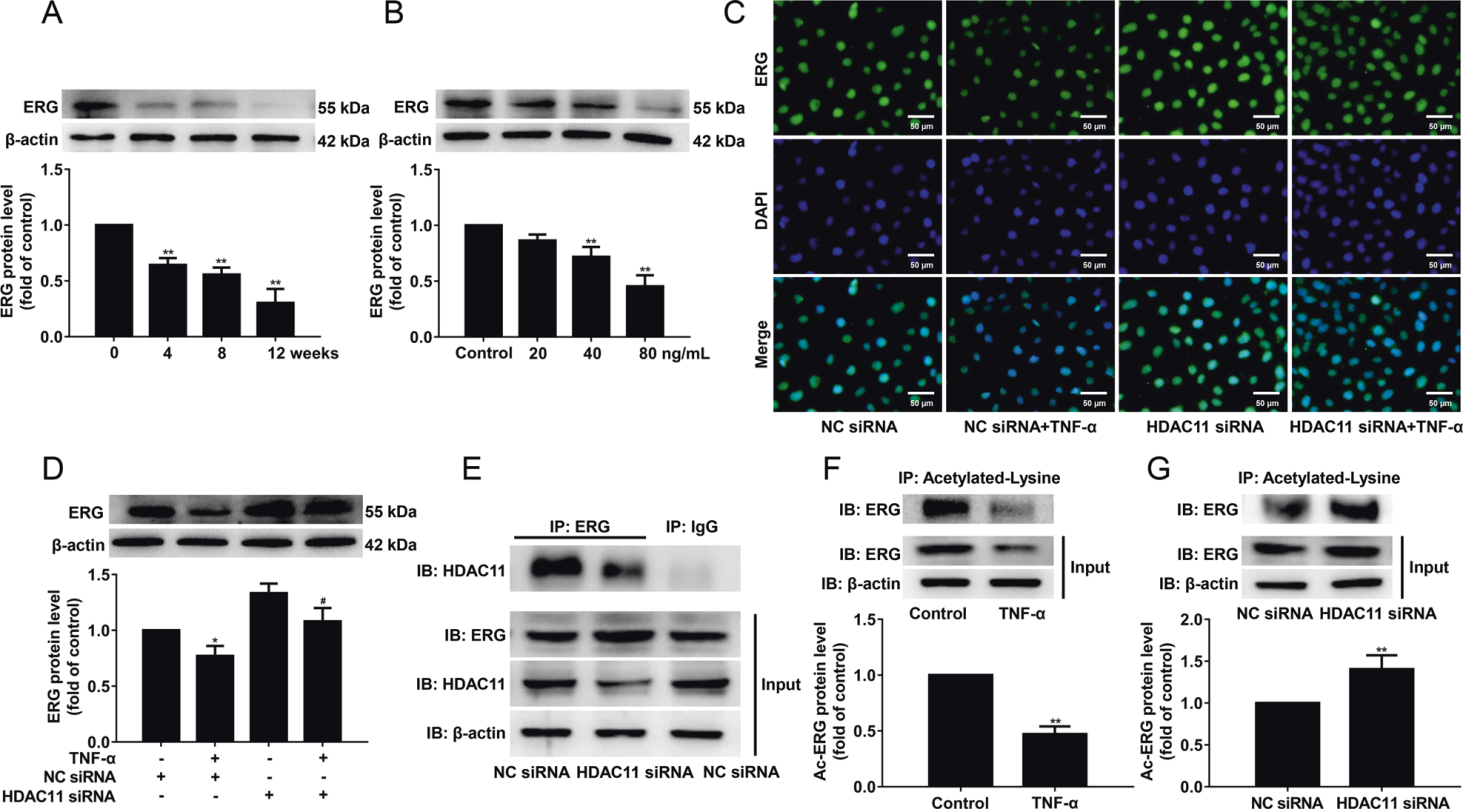
HDAC11 formes a complex with ERG and decreases ERG acetylation in HUVECs. **A** ApoE^−/−^ mice were fed an HFD for 0, 4, 8, or 12 weeks. ERG protein expression was determined by Western blotting (*n* = 3). **P < 0.01 vs. ND group. **B** HUVECs were treated with TNF-α (20, 40, or 80 ng/mL) for 12 h. ERG protein expression was determined by Western blotting. HUVECs were stimulated with 40 ng/mL TNF-α for 12 h after their transfection with HDAC11 siRNA or NC siRNA for 48 h. **C**, **D** The expression and localization of ERG were determined by Western blotting and Immunofluorescence staining, ×200. Scale bar indicates 50 μm. **E** HUVECs were transfected with HDAC11 siRNA or NC siRNA for 48 h. The protein lysates were precipitated with ERG or IgG antibody and then were analyzed by Western blotting. **F** HUVECs were treated with TNF-α for 12 h. The protein lysates were precipitated with acetylated-lysine antibody and then were analyzed by Western blotting. **G** HUVECs were transfected with HDAC11 siRNA or NC siRNA for 48 h. The protein lysates of HUVECs transfected with HDAC11 siRNA or NC siRNA were precipitated with acetylated-lysine antibody and then were analyzed by Western blotting. *P < 0.05, **P < 0.01 vs. control group or NC siRNA; ^#^*P* < 0.05 vs. NC siRNA + TNF-α group. Results are expressed as mean ± SD (*n* = 3).

As shown in Fig. 7A, ERG protein expression was significantly decreased in the aorta of ApoE^−/−^ mice after HFD feeding for 4 weeks. Meanwhile, we found that TNF-α significantly decreased ERG protein expression (Fig. 7B). To investigate whether ERG is regulated by HDAC11, HUVECs were transfected with HDAC11 siRNA or NC siRNA and exposed to TNF-α. We found that HDAC11 knockdown significantly increased ERG protein expression (Fig. 7C, D). To further test this, immunoprecipitation was performed with HUVECs transfected with HDAC11 siRNA or NC siRNA. The results showed that HDAC11 knockdown suppressed the formation of complexes of HDAC11 and ERG (Fig. 7E). To investigate whether HDAC11 alters the acetylation status of ERG, the protein lysates of HUVECs transfected with HDAC11 siRNA or NC siRNA were precipitated with acetylated-lysine antibodies and then were analyzed by Western blotting. The results showed that TNF-α significantly decreased acetylated ERG, while HDAC11 knockdown markedly increased acetylated ERG compared with the NC siRNA group (Fig. 7F, G).

**Fig. 8:**
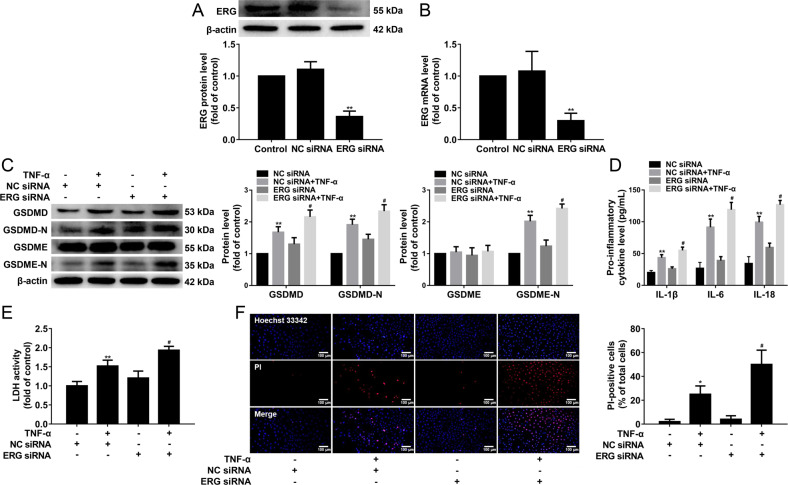
ERG knockdown further augmented pyroptosis in TNF-α-induced HUVECs. **A**, **B** HUVECs were transfected with ERG siRNA or NC siRNA for 48 h, and the protein and mRNA expressions of ERG were determined by western blotting and quantitative real-time PCR. **C** HUVECs were stimulated with 40 ng/mL TNF-α for 12 h after their transfection with ERG siRNA or NC siRNA. The protein expressions of GSDMD, GSDMD-N, GSDME, and GSDME-N were determined by Western blotting. **D** The levels of IL-1β, IL-6, and IL-18 in the cellular supernatant were detected by ELISA assay. **E** The cellular supernatant LDH level was evaluated with a cytotoxicity detection LDH kit. **F** The representative photographs of double-fluorescent staining with PI (red) and Hoechst 33342 (blue), and the quantification of PI-positive cells, ×200. Scale bar indicates 100 μm. *P < 0.05, **P < 0.01 vs. NC siRNA group; ^#^*P* < 0.05 *vs* NC siRNA + TNF-α group. Results are expressed as mean ± SD (*n* = 3).

Based on the above results, we inferred that HDAC11 regulated endothelial cell pyroptosis through ERG. To test this, HUVECs were transfected with ERG siRNA or NC siRNA for 48 h. Our results showed that the efficiency of ERG silencing in HUVECs was approximately 64% at the protein level and 70% at the mRNA level (Fig. 8A, B). We found that ERG knockdown further increased GSDMD-N and GSDME-N protein expressions (Fig. 8C), and further promoted the release of IL-1β, IL-6, and IL-18 (Fig. 8D). ERG knockdown also further increased the cellular supernatant LDH activity and the number of PI-positive cells (Fig. 8E, F). These results suggested that HDAC11 might regulate pyroptosis via ERG in TNF-α-induced HUVECs.

## Discussion

This study provided evidence that HDAC11 is highly expressed in the aortic intima of HFD-fed ApoE^−/−^ mice and HDAC11 knockdown significantly suppressed pyroptosis via inhibiting NLRP3/caspase-1/GSDMD and caspase-3/GSDME pathways in TNF-α-induced HUVECs. Importantly, HDAC11 formed a complex with ERG and decreased the acetylation levels of ERG, and subsequently promoted pyroptosis (summarized in Fig. S2). Our study unraveled pyroptosis as a cellular mechanism for the pro-inflammatory property of HDAC11, thereby advancing our understanding of promoting effects of HDAC11 on the endothelial cell pyroptosis and inflammatory response in AS process.

HDAC enzymes regulate diverse biological functions, including gene expression, rendering them potential targets for intervention in several diseases [21]. Among the human zinc-dependent HDACs, the most recently discovered member, HDAC11, has been shown to play a role in cardiovascular diseases through promoting inflammation [3–5]. Recently, it has been found that HDAC11 protein expression was significantly elevated both in human atherosclerotic tissue samples and in the atherosclerotic aorta of ApoE^−/−^ mice [6]. Our present results also showed that HDAC11 expression was significantly increased in the aorta of HFD-fed ApoE^−/−^ mice. The process of pyroptosis is characterized predominantly by the formation of gasdermin protein family mediated membrane perforation, cell collapse, and the release of pro-inflammatory cytokines such as IL-1β and IL-18 [22]. It has been reported that GSDMD and GSDME were found to be expressed in the artery [15]. Our study demonstrated that HFD feeding results in NLRP3 inflammasome, caspase-1, and caspase-3 activation, and subsequent GSDMD and GSDME cleavage in the aorta of ApoE^−/−^ mice. Therefore, HDAC11 might participate in the regulation of endothelial cell pyroptosis in the process of AS.

TNF-α is an important inflammatory mediator closely related to the occurrence and development of AS [23]. The study found that TNF-α increased the activity of NLRP3 inflammasome in CACO2 cells [24]. In addition, TNF-α induced caspase-1 expression and activation in A549 cells and 3T3-L1 cells [25, 26]. During pyroptosis, the NLRP3 inflammasome activates the caspase-1 precursor and then mediates GSDMD cleavage and maturation of IL-1β and IL-18 [27]. In the current study, our data demonstrated that TNF-α activated NLRP3 inflammasome and caspase-1, and further induced GSDMD-mediated pyroptosis in HUVECs. Moreover, a large number of studies have shown that TNF-α can induce the activation of caspase-3 and then induce the occurrence of apoptosis in endothelial cells [28–30]. Pyroptosis and intrinsic apoptosis are two forms of regulated cell death driven by active caspases. Caspase-1 induces GSDMD pore formation during pyroptosis, whereas caspase-3 promotes GSDME pore formation during apoptosis [31]. Our results showed that TNF-α activated caspase-3, and further induced GSDME-mediated pyroptosis in HUVECs. It has been reported that TNF-α can upregulate the expression of HDAC11 in B cells [8]. Our present results revealed that TNF-α can upregulate HDAC11 expression in HUVECs. It has been shown that TNF-α induced necroptosis by its cognate receptor TNFR1 [32]. Our study suggested that TNF-α induced pyroptosis and upregulated HDAC11 expression via TNFR1 in HUVECs. In addition, a study showed that HDAC inhibitor Trichostatin A mitigated the inflammation-induced pyroptosis during endotoxemia-induced acute lung injury [33]. In the present study, HDAC11 knockdown significantly inhibited pyroptosis. The inhibition of endothelial cell pyroptosis is of great significance to prevent AS [34]. Disulfiram and NSA can directly affect GSDMD pore formation to inhibit pyroptosis [35, 36]. In addition, GSDMD knockdown can attenuate high-glucose-induced inflammation and apoptosis in podocytes [37]. GSDME knockdown attenuated ATP-induced cell death and HMGB1 release in RAW264.7 cells [38]. Our present study found that GSDME knockdown significantly decreased TNF-α-induced pyroptosis and inflammatory response, while disulfiram and NSA further augmented the inhibitory effects of GSDME knockdown on pyroptosis and inflammatory response. These results confirmed that HDAC11 promoted pyroptosis via NLRP3/caspase-1/GSDMD and caspase-3/GSDME pathways in TNF-α-induced HUVECs.

ERG remains highly expressed in endothelial cells of most adult tissues, which acts as a key regulator of endothelial homeostasis [19, 39]. A previous study showed that ERG expression was lost in human coronary plaques from the endothelium overlaying active plaque shoulders [20]. We observed a marked reduction of ERG protein expression in the aorta of HFD-fed ApoE^−/−^ mice. It has been shown that inflammatory stimuli such as TNF-α can down-regulate ERG expression in HUVECs [40]. We also observed that TNF-α significantly decreased ERG protein expression in HUVECs. In addition, it has been unraveled that HDAC11 formed complexes with ERG in HUVECs [5]. In our present results, we observed that HDAC11 knockdown significantly increased ERG protein expression, and suppressed the formation of complexes of HDAC11 and ERG. Moreover, previous results found that PAR2AP significantly decreased acetylated ERG in HUVECs [5]. Our present results showed that TNF-α significantly decreased acetylated ERG, while HDAC11 knockdown markedly increased acetylated ERG in HUVECs. ERG inhibition resulted in a significant decrease in vascularization with the increase in caspase-positive endothelial cells [41]. In addition, ERG inhibited NF-κB dependent transcription and inflammation in mice [20]. In the present study, the data revealed that ERG knockdown further augmented TNF-α-induced pyroptosis, as evidenced by further cleavage of GSDMD and GSDME, the further release of IL-1β, IL-6, and IL-18, as well as further elevation of LDH activity and increase of PI-positive cells. These results further confirmed that HDAC11 formed a complex with ERG and decreased ERG acetylation to regulate pyroptosis in TNF-α-induced HUVECs.

Collectively, our results provided evidence that HDAC11 promoted both NLRP3/caspase-1/GSDMD and caspase-3/GSDME pathways leading to vascular endothelial cell pyroptosis via regulation of ERG in TNF-α-induced HUVECs. Hence, pyroptosis is likely a cellular mechanism underlying the pro-inflammatory effect of HDAC11 in AS process. These findings might be pivotal for understanding the potential role of HDAC11 in vascular endothelial cell pyroptosis and AS.

## Supplementary information

Supplementary Figure Legends

Table S1 Primer sequences used in real-time PCR

Fig. S1. HFD feeding increases NLRP3, ASC, cleaved caspase-1 and cleaved caspase-3 protein expression in the aorta of ApoE-/- mice.

Fig. S2. Schematic illustration of the signaling pathway involved in the effect of HDAC11 on pyroptosis in HUVECs.

Original Data File

## Data Availability

Data supporting present findings are available from the corresponding author upon reasonable request.

## References

[1] Villagra A, Cheng F, Wang HW, Suarez I, Glozak M, Maurin M (2009). The histone deacetylase HDAC11 regulates the expression of interleukin 10 and immune tolerance. Nat Immunol.

[2] Leslie PL, Chao YL, Tsai YH, Ghosh SK, Porrello A, Van Swearingen AED (2019). Histone deacetylase 11 inhibition promotes breast cancer metastasis from lymph nodes. Nat Commun.

[3] Fan XD, Wan LL, Duan M, Lu S (2018). HDAC11 deletion reduces fructose-induced cardiac dyslipidemia, apoptosis and inflammation by attenuating oxidative stress injury. Biochem Biophys Res Commun.

[4] Zhou B, Zeng S, Li N, Yu L, Yang G, Yang Y (2017). Angiogenic factor with G patch and FHA domains 1 is a novel regulator of vascular injury. Arterioscler Thromb Vasc Biol.

[5] Zhang R, Ge J (2017). Proteinase-activated receptor-2 modulates Ve-cadherin expression to affect human vascular endothelial barrier function. J Cell Biochem.

[6] Manea SA, Vlad ML, Fenyo IM, Lazar AG, Raicu M, Muresian H (2020). Pharmacological inhibition of histone deacetylase reduces NADPH oxidase expression, oxidative stress and the progression of atherosclerotic lesions in hypercholesterolemic apolipoprotein E-deficient mice; potential implications for human atherosclerosis. Redox Biol.

[7] Yuan L, Chen X, Cheng L, Rao M, Chen K, Zhang N (2018). HDAC11 regulates interleukin-13 expression in CD4+ T cells in the heart. J Mol Cell Cardiol.

[8] Shao JB, Luo XQ, Wu YJ, Li MG, Hong JY, Mo LH (2018). Histone deacetylase 11 inhibits interleukin 10 in B cells of subjects with allergic rhinitis. Int Forum Allergy Rhinol.

[9] Xu YJ, Zheng L, Hu YW, Wang Q (2018). Pyroptosis and its relationship to atherosclerosis. Clin Chim Acta.

[10] Oh S, Son M, Park CH, Jang JT, Son KH, Byun K (2020). The reducing effects of pyrogallol-phloroglucinol-6,6-bieckol on high-fat diet-induced pyroptosis in endothelial and vascular smooth muscle cells of mice aortas. Mar Drugs.

[11] De Schutter E, Roelandt R, Riquet FB, Van Camp G, Wullaert A, Vandenabeele P (2021). Punching holes in cellular membranes: biology and evolution of gasdermins. Trends Cell Biol.

[12] Liu X, Zhang Z, Ruan J, Pan Y, Magupalli VG, Wu H (2016). Inflammasome-activated gasdermin D causes pyroptosis by forming membrane pores. Nature.

[13] Wu X, Zhang H, Qi W, Zhang Y, Li J, Li Z (2018). Nicotine promotes atherosclerosis via ROS-NLRP3-mediated endothelial cell pyroptosis. Cell Death Dis.

[14] Wu P, Chen J, Chen J, Tao J, Wu S, Xu G (2020). Trimethylamine N-oxide promotes apoE−/− mice atherosclerosis by inducing vascular endothelial cell pyroptosis via the SDHB/ROS pathway. J. Cell Physiol.

[15] Broz P, Pelegrín P, Shao F (2020). The gasdermins, a protein family executing cell death and inflammation. Nat Rev Immunol.

[16] Rogers C, Erkes DA, Nardone A, Aplin AE, Fernandes-Alnemri T, Alnemri ES (2019). Gasdermin pores permeabilize mitochondria to augment caspase-3 activation during apoptosis and inflammasome activation. Nat Commun.

[17] Shah AV, Birdsey GM, Randi AM (2016). Regulation of endothelial homeostasis, vascular development and angiogenesis by the transcription factor ERG. Vasc Pharm.

[18] Kalna V, Yang Y, Peghaire CR, Frudd K, Hannah R, Shah AV (2019). The transcription factor ERG regulates super-enhancers associated with an endothelial-specific gene expression program. Circ Res.

[19] Yuan L, Le BA, Sacharidou A, Itagaki K, Zhan Y, Kondo M (2012). ETS-related gene (ERG) controls endothelial cell permeability via transcriptional regulation of the claudin 5 (CLDN5) gene. J Biol Chem.

[20] Sperone A, Dryden NH, Birdsey GM, Madden L, Johns M, Evans PC (2011). The transcription factor Erg inhibits vascular inflammation by repressing NF-kappaB activation and proinflammatory gene expression in endothelial cells. Arterioscler Thromb Vasc Biol.

[21] Moreno-Yruela C, Galleano I, Madsen AS, Olsen CA (2018). Histone deacetylase 11 Is an ε-N-myristoyllysine hydrolase. Cell Chem Biol.

[22] Zhang KJ, Wu Q, Jiang SM, Ding L, Liu CX, Xu M (2021). Pyroptosis: a new frontier in kidney diseases. Oxid Med Cell Longev.

[23] Ha SJ, Lee J, Song KM, Kim YH, Lee NH, Kim YE (2018). Ultrasonicated Lespedeza cuneata extract prevents TNF-α-induced early atherosclerosis in vitro and in vivo. Food Funct.

[24] Tao Z, Zhou X, Zhang Y, Pu W, Yang Y, Wei F (2021). Attenuates dextran sulfate sodium-induced colitis in rats and TNF-α-stimulated colitis in CACO2 cells: involvement of the NLRP3 inflammasome and autophagy. Media Inflamm.

[25] Jain N, Ch S, Swarup G (2007). Tumor necrosis factor-alpha-induced caspase-1 gene expression role p73. FEBS J.

[26] Furuoka M, Ozaki K, Sadatomi D, Mamiya S, Yonezawa T, Tanimura S (2016). TNF-α induces caspase-1 activation independently of simultaneously induced NLRP3 in 3T3-L1 cells. J Cell Physiol.

[27] Zhao W, Yang H, Lyu L, Zhang J, Xu Q, Jiang N (2020). GSDMD, an executor of pyroptosis, is involved in IL-1β secretion in *Aspergillus fumigatus* keratitis. Exp Eye Res.

[28] Yu J, Ma M, Ma Z, Fu J (2016). HDAC6 inhibition prevents TNF-α-induced caspase 3 activation in lung endothelial cell and maintains cell-cell junctions. Oncotarget.

[29] Zhou P, Lu S, Luo Y, Wang S, Yang K, Zhai Y (2017). Attenuation of TNF-α-induced inflammatory injury in endothelial cells by ginsenoside Rb1 via inhibiting NF-κB, JNK and p38. Front Pharm.

[30] Oita RC, Camp SM, Ma W, Ceco E, Harbeck M, Singleton P (2018). Novel mechanism for nicotinamide phosphoribosyltransferase inhibition of TNF-α-mediated apoptosis in human lung endothelial cells. Am J Respir Cell Mol Biol.

[31] de Torre-Minguela C, Gómez AI, Couillin I, Pelegrín P (2021). Gasdermins mediate cellular release of mitochondrial DNA during pyroptosis and apoptosis. FASEB J.

[32] Hanson B (2016). Necroptosis: a new way of dying. Cancer Biol Ther.

[33] Samanta S, Zhou Z, Rajasingh S, Panda A, Sampath V, Rajasingh J (2018). DNMT and HDAC inhibitors together abrogate endotoxemia mediated macrophage death by STAT3-JMJD3 signaling. Int J Biochem Cell Biol.

[34] Zhang Y, Liu X, Bai X, Lin Y, Li Z, Fu J (2018). Melatonin prevents endothelial cell pyroptosis via regulation of long noncoding RNA MEG3/miR-223/NLRP3 axis. J Pineal Res.

[35] Hu JJ, Liu X, Xia S, Zhang Z, Zhang Y, Zhao J (2020). FDA-approved disulfiram inhibits pyroptosis by blocking gasdermin D pore formation. Nat Immunol.

[36] Rathkey JK, Zhao J, Liu Z, Chen Y, Yang J, Kondolf HC (2018). Chemical disruption of the pyroptotic pore-forming protein gasdermin D inhibits inflammatory cell death and sepsis. Sci Immunol.

[37] Li H, Zhao K, Li Y (2021). Gasdermin D protects mouse podocytes against high-glucose-induced inflammation and apoptosis via the C-Jun N-terminal kinase (JNK) pathway. Med Sci Monit.

[38] Zeng CY, Li CG, Shu JX, Xu LH, Ouyang DY, Mai FY (2019). ATP induces caspase-3/gasdermin E-mediated pyroptosis in NLRP3 pathway-blocked murine macrophages. Apoptosis.

[39] Zhang X, Hu C, Yuan YP, Song P, Kong CY, Wu HM (2021). Endothelial ERG alleviates cardiac fibrosis via blocking endothelin-1-dependent paracrine mechanism. Cell Biol Toxicol.

[40] Yuan L, Nikolova-Krstevski V, Zhan Y, Kondo M, Bhasin M, Varghese L (2009). Antiinflammatory effects of the ETS factor ERG in endothelial cells are mediated through transcriptional repression of the interleukin-8 gene. Circ Res.

[41] Birdsey GM, Dryden NH, Amsellem V, Gebhardt F, Sahnan K, Haskard DO (2008). Transcription factor Erg regulates angiogenesis and endothelial apoptosis through VE-cadherin. Blood.

