# Supplementary material for: HDAC11 promotes both NLRP3/caspase-1/GSDMD and caspase-3/GSDME pathways causing pyroptosis via ERG in vascular endothelial cells
Source: Cell Death Discov. 2022 Mar 12;8:112. doi: 10.1038/s41420-022-00906-9 (PMC8918356; doi:10.1038/s41420-022-00906-9)
Supplement: Supplementary file 1 — Supplementary Figure Legends [file 41420_2022_906_MOESM1_ESM.doc]

**Supplementary Figure Legends**

**Fig. S1. HFD feeding increases NLRP3, ASC, cleaved caspase-1 and cleaved caspase-3 protein expression in the aorta of ApoE^-/-^ mice.** (A) ApoE^-/-^ mice were fed a HFD for 0, 4, 8 or 12 weeks. The expressions of NLRP3, ASC, pro-caspase-1, cleaved caspase-1, pro-caspase-3 and cleaved caspase-3 in the aorta of ApoE^-/-^ mice were determined by Western blotting (n=3). (B) The expression of cleaved caspase-1 in the aortic intima by immunofluorescent double staining of the aortic sinus of Cleaved-caspase-1 and CD31. The nuclei were stained blue with DAPI. Scale bar indicates 50 μm. **P* < 0.05, ***P* < 0.01 *vs* ND group.

**Fig. S2. Schematic illustration of the signaling pathway involved in the effect of HDAC11 on pyroptosis in HUVECs.**

**Table S1. Primer sequences used in real-time PCR.**
