# Supplementary material for: HDAC11 promotes both NLRP3/caspase-1/GSDMD and caspase-3/GSDME pathways causing pyroptosis via ERG in vascular endothelial cells
Source: Cell Death Discov. 2022 Mar 12;8:112. doi: 10.1038/s41420-022-00906-9 (PMC8918356; doi:10.1038/s41420-022-00906-9)
Supplement: Supplementary file 2 — Table S1 Primer sequences used in real-time PCR [file 41420_2022_906_MOESM2_ESM.doc]

**Table S**1 Primer sequences used in real-time PCR

| Gene | Primer | Sequence (5’-3’) |
| --- | --- | --- |
| Mouse HDAC11 | F | GCCCTGGATCTGCTCCAACTAC |
|  | R | AACTGGTGACTCTGGCATCCTC |
| Mouse GAPDH | F | TGTGTCCGTCGTGGATCTGA |
|  | R | TTGCTGTTGAAGTCGCAGGAG |
| Human HDAC11 | F | GATGTCTACAACCGCCACATCTACC |
|  | R | CCTGCATTGTATACCACCACGTC |
| Human NLRP3 | F | CACCTGTTGTGCAATCTGAAG |
|  | R | GCAAGATCCTGACAACATGC |
| Human TNFR1 | F | CTGCCAGGAGAAACAGAACA |
|  | R | CTGGAGGTGAAGGTGGAACT |
| Human TNFR2 | F | TGAAACATCAGACGTGGTGTG |
|  | R | TGCAAATATCCGTGGATGAAGTC |
| Human IL-1β | F | CTGTGTCTTTCCCGTGGACC |
|  | R | CAGCTCATATGGGTCCGACA |
| Human IL-6 | F | CCTGAACCTTCCAAAGATGGC |
|  | R | TTCACCAGGCAAGTCTCCTCA |
| Human MCP-1 | F | CAGCCAGATGCAATCAATGCC |
|  | R | TGGAATCCTGAACCCACTTCT |
| Human GAPDH | F | GGAGCGAGATCCCTCCAAAAT |
|  | R | GGCTGTTGTCATACTTCTCATGG |
