# Supplementary figures and images for: HDAC11 promotes both NLRP3/caspase-1/GSDMD and caspase-3/GSDME pathways causing pyroptosis via ERG in vascular endothelial cells
Source: Cell Death Discov. 2022 Mar 12;8:112. doi: 10.1038/s41420-022-00906-9 (PMC8918356; doi:10.1038/s41420-022-00906-9)

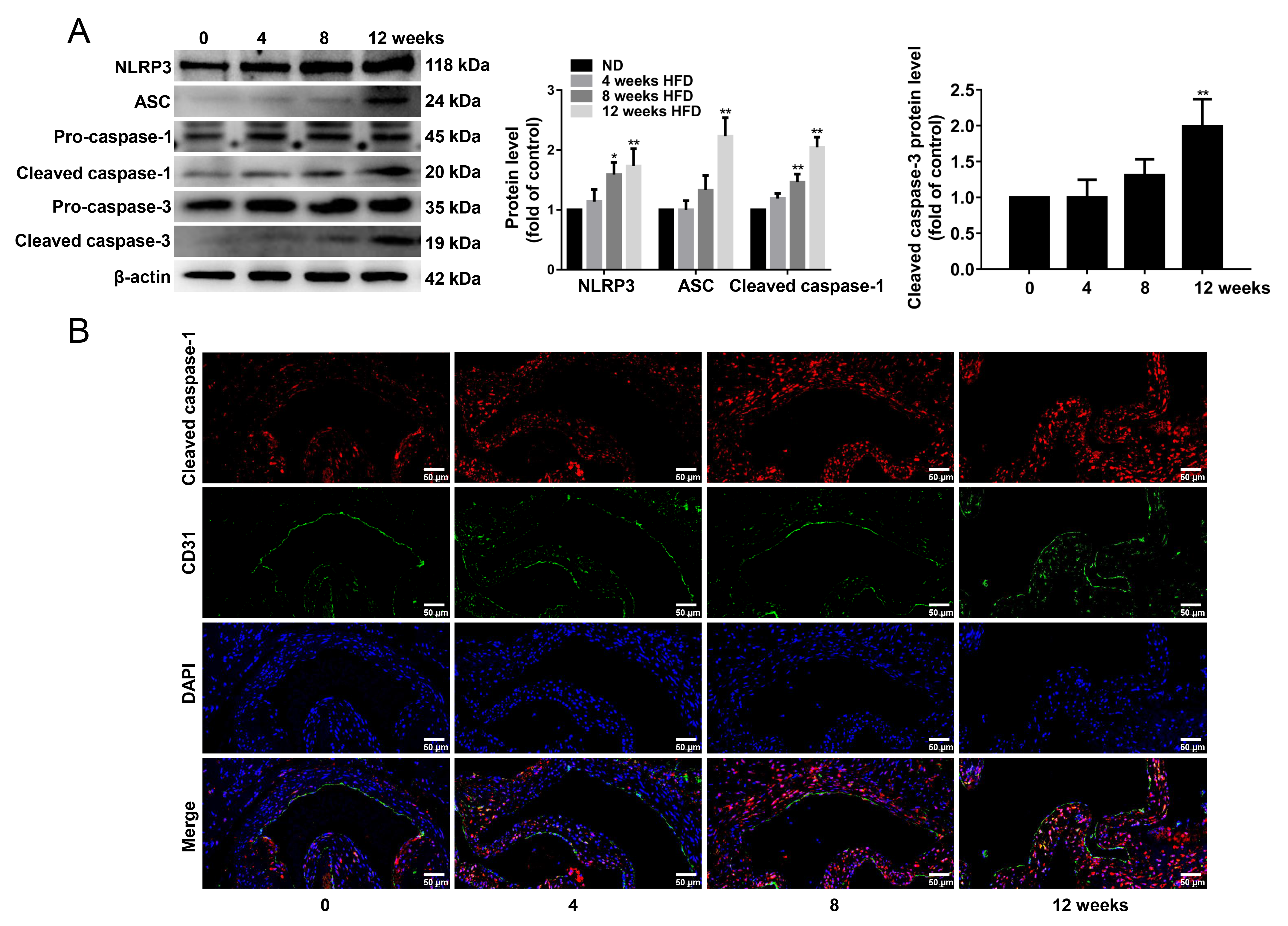

Supplement: Supplementary file 3 — Fig. S1. HFD feeding increases NLRP3, ASC, cleaved caspase-1 and cleaved caspase-3 protein expression in the aorta of ApoE-/- mice. [file 41420_2022_906_MOESM3_ESM.tif]

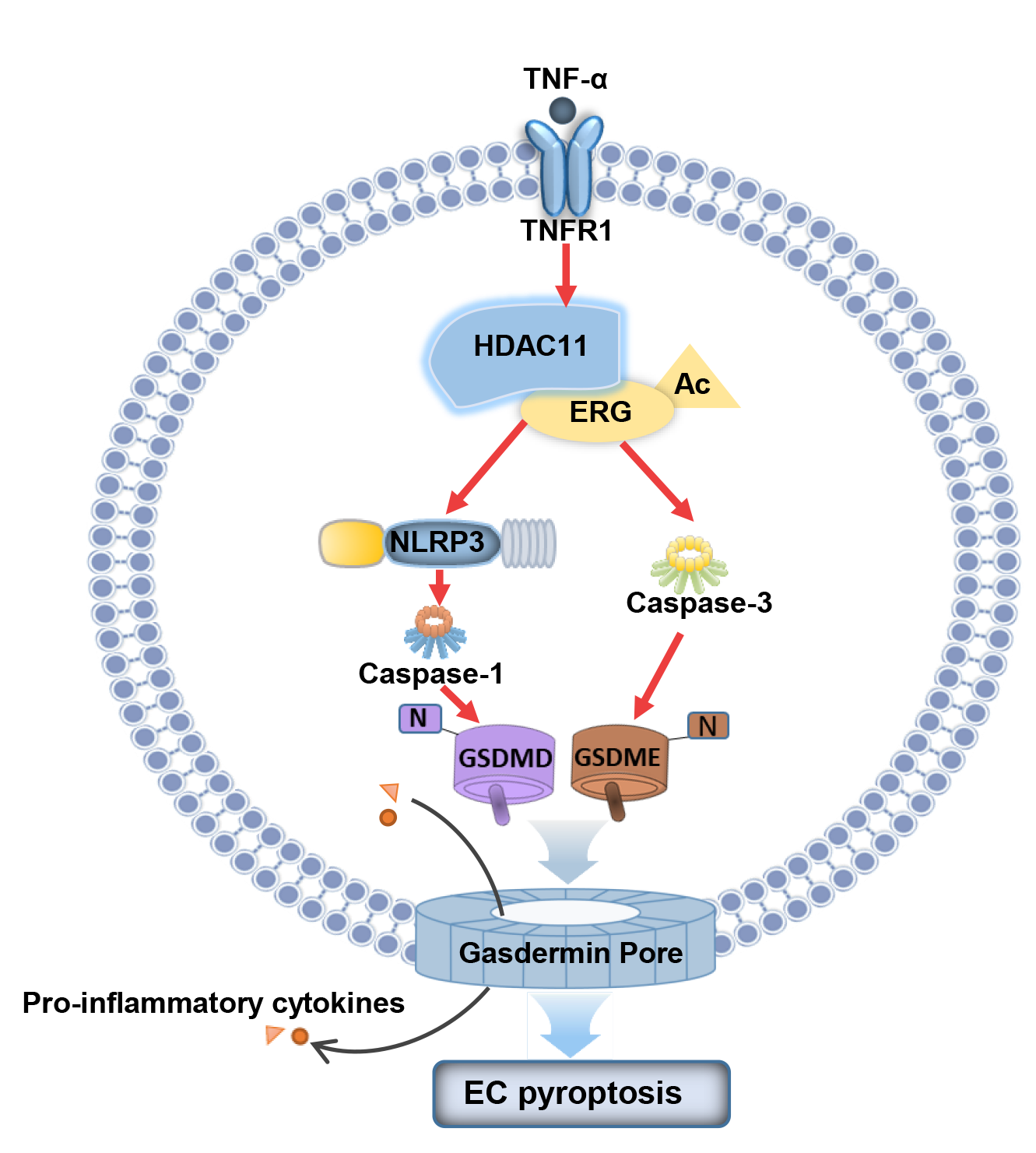

Supplement: Supplementary file 4 — Fig. S2. Schematic illustration of the signaling pathway involved in the effect of HDAC11 on pyroptosis in HUVECs. [file 41420_2022_906_MOESM4_ESM.tif]

Western blotting

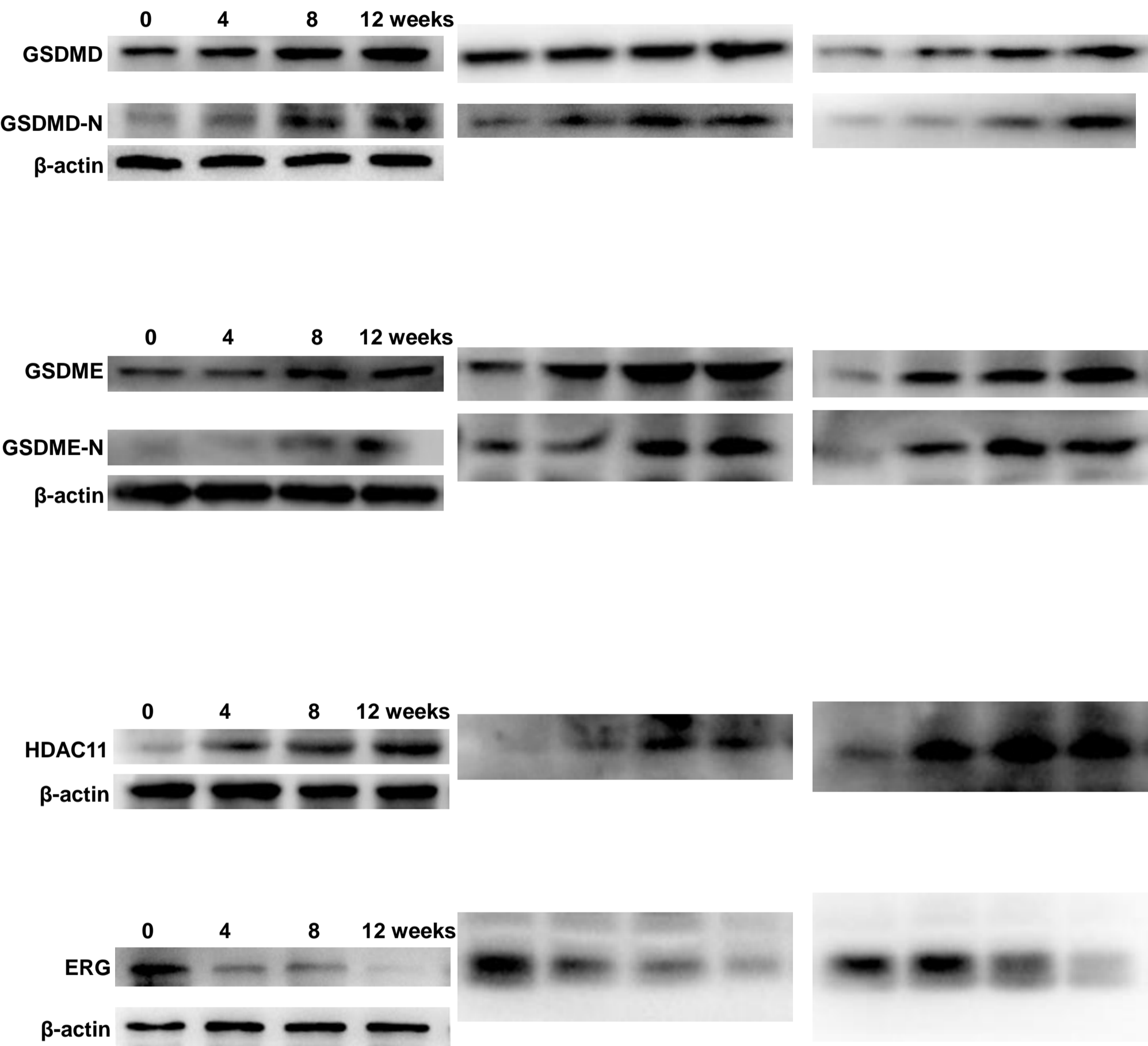

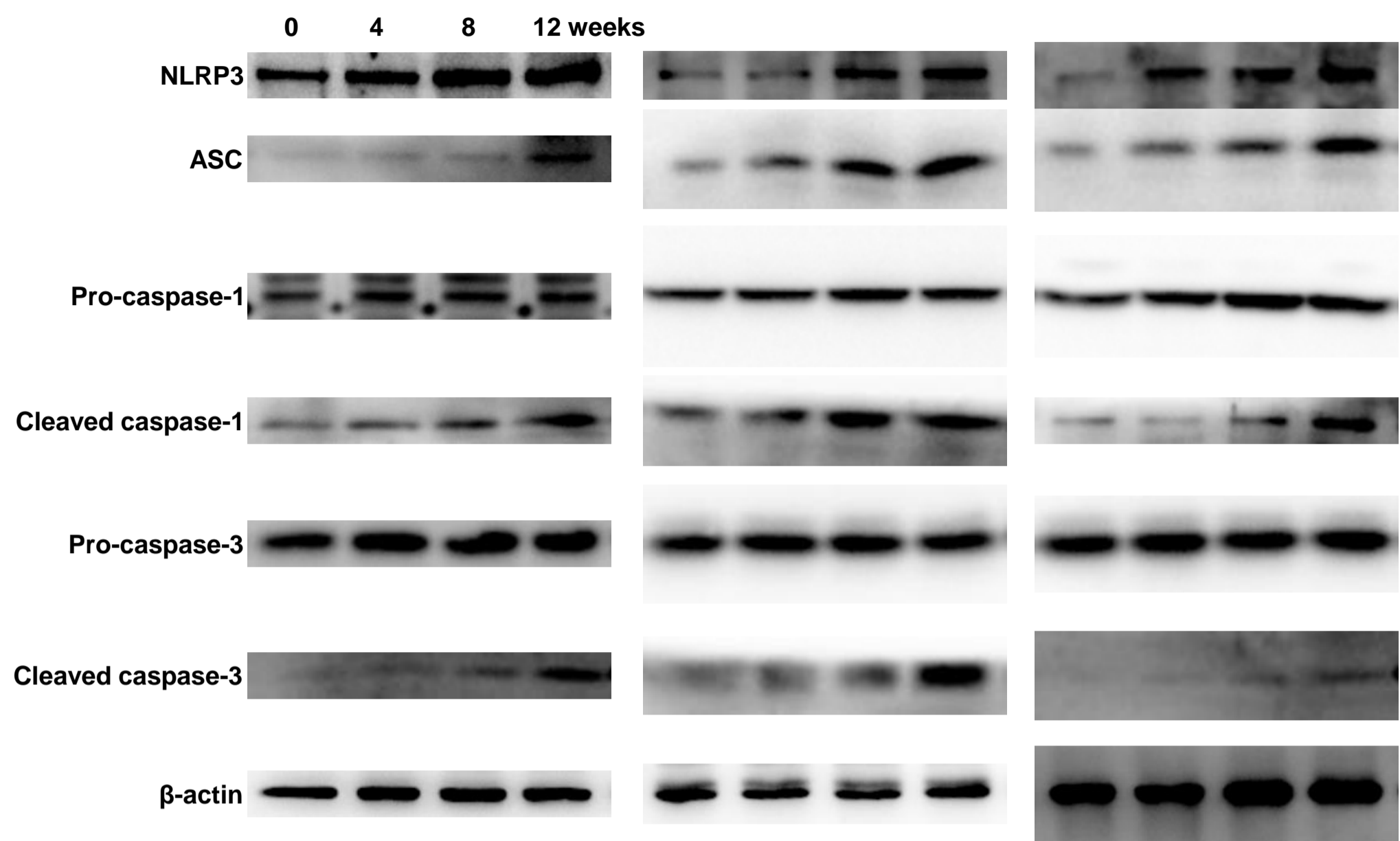

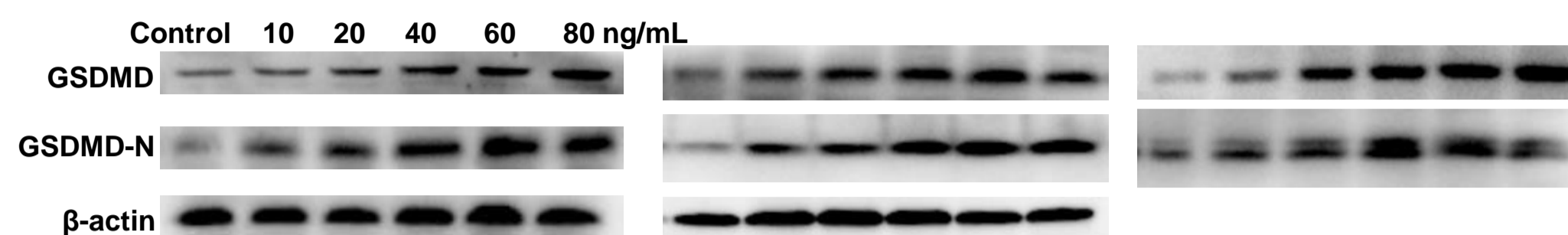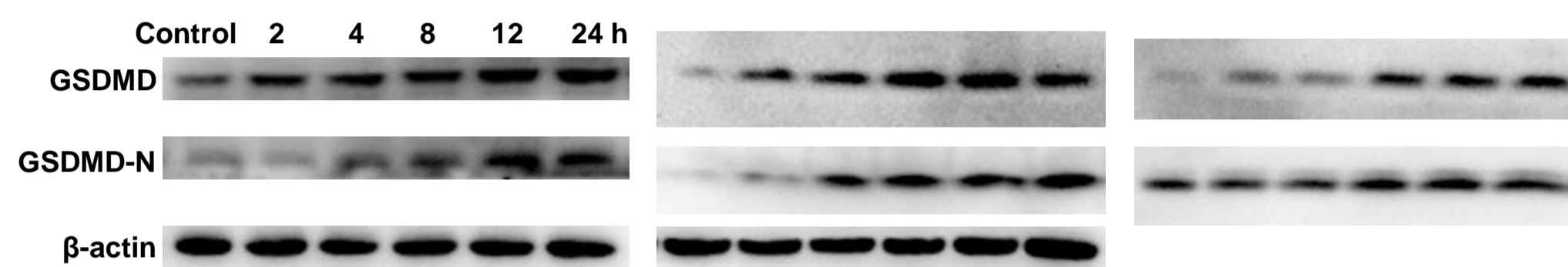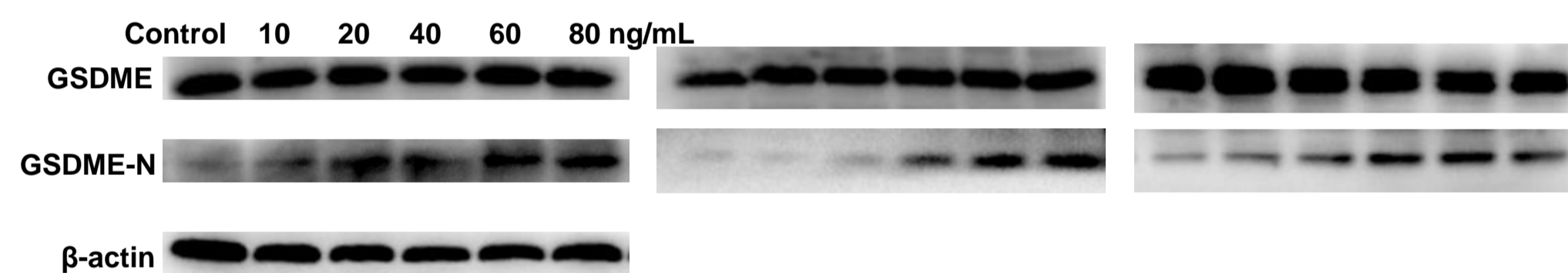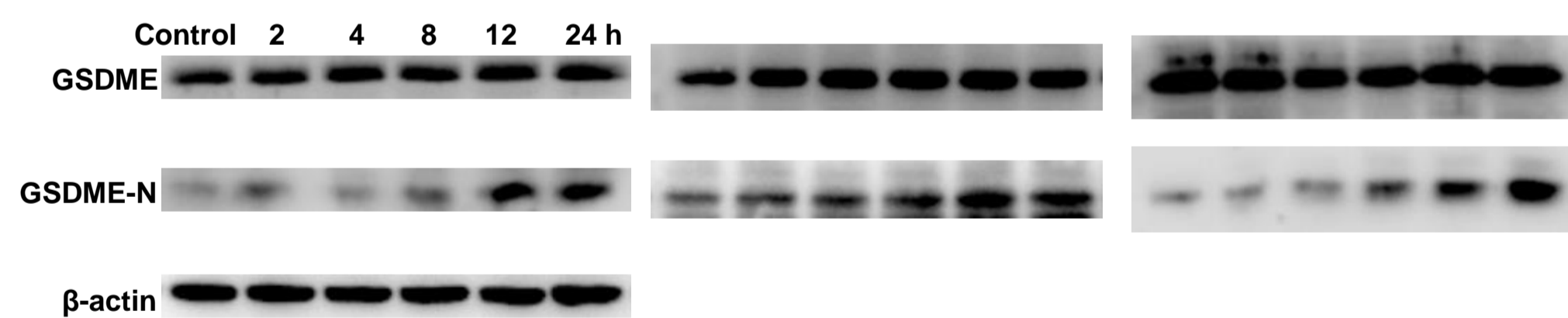

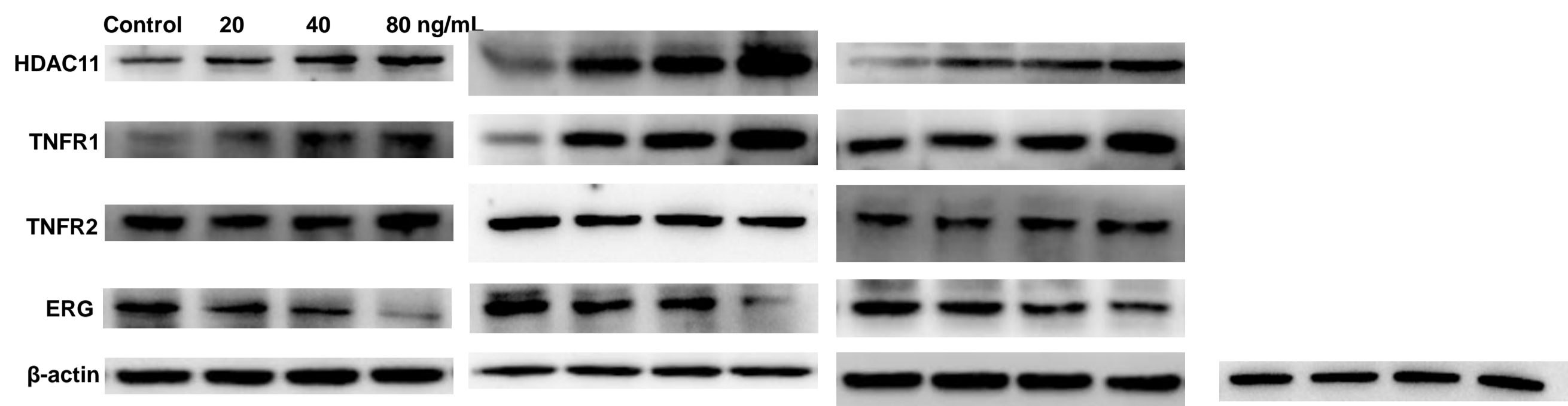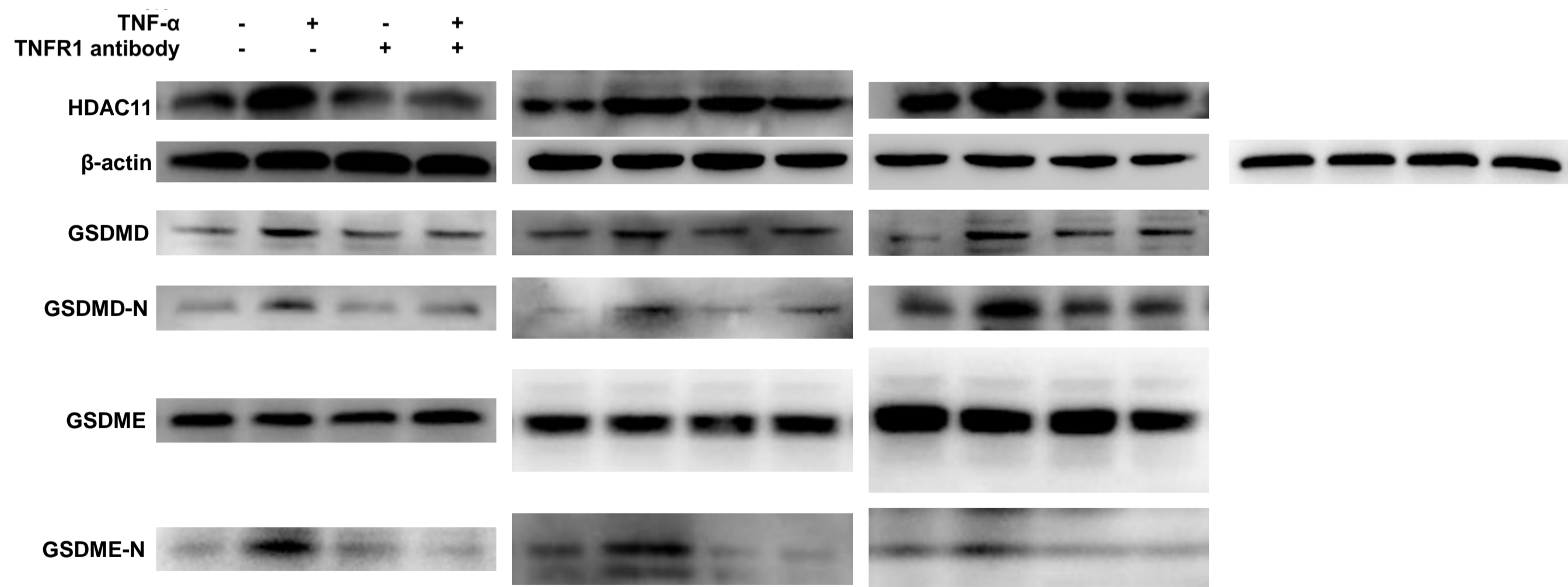

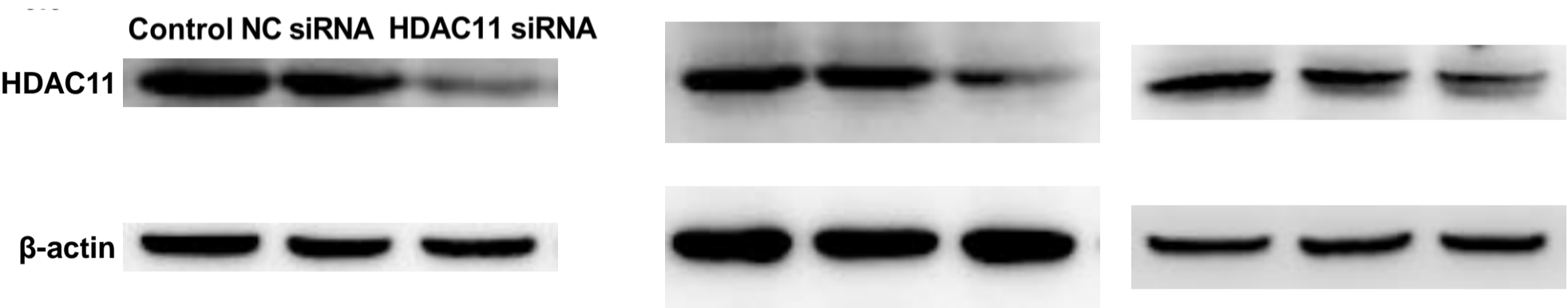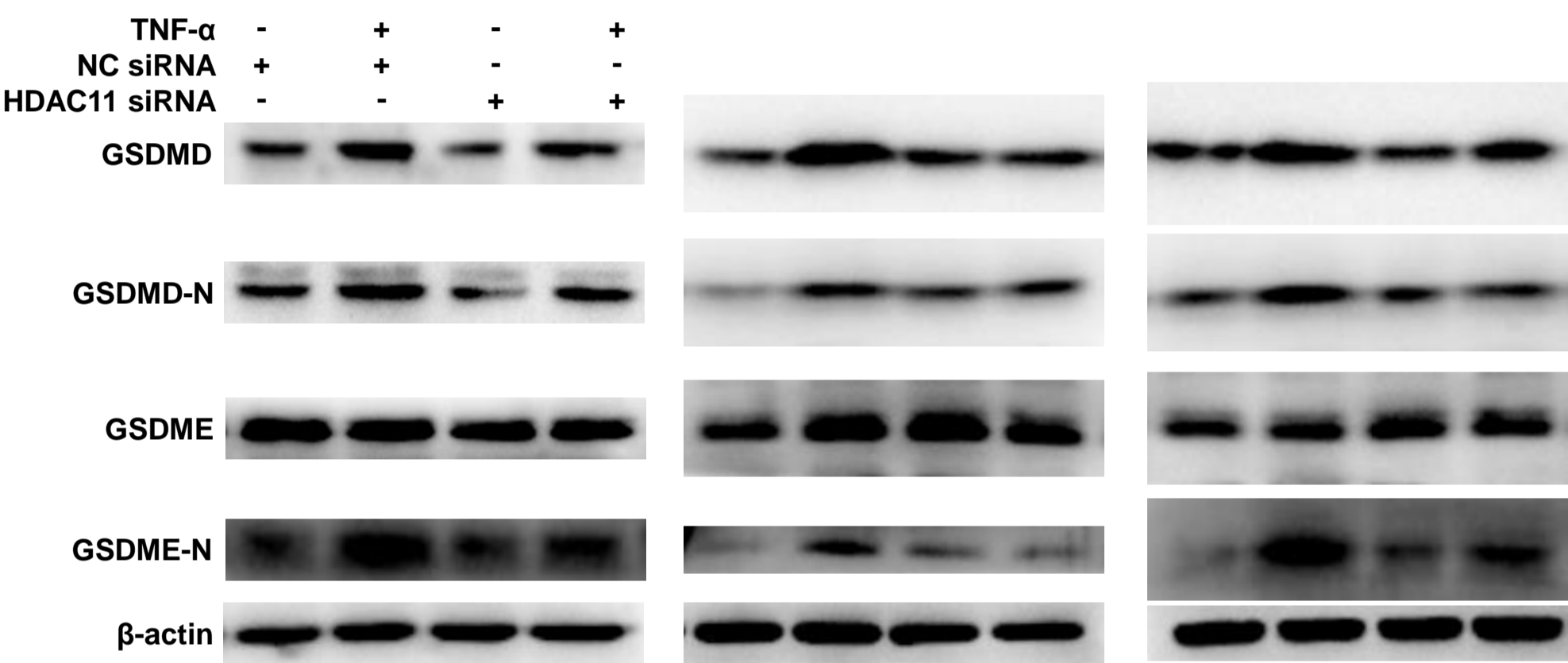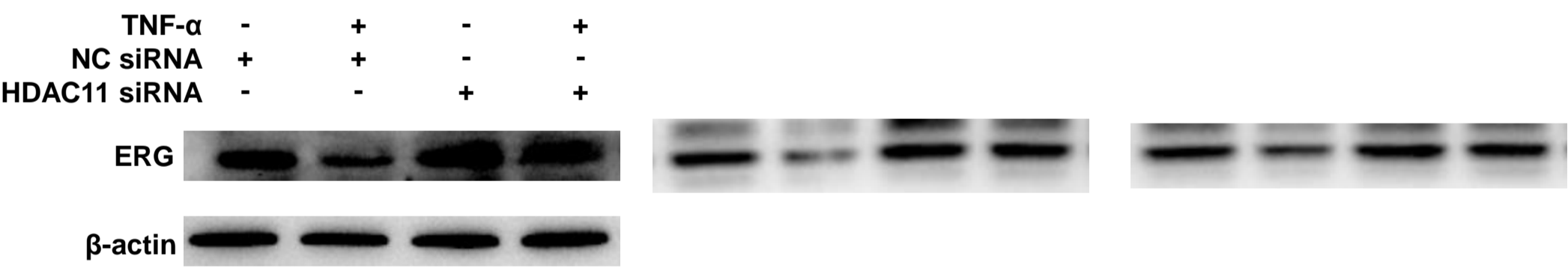

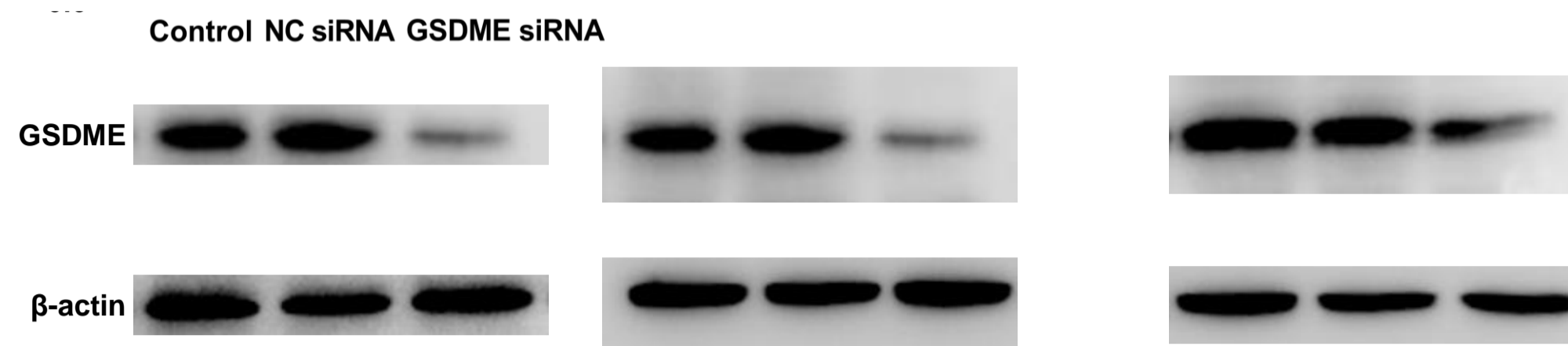

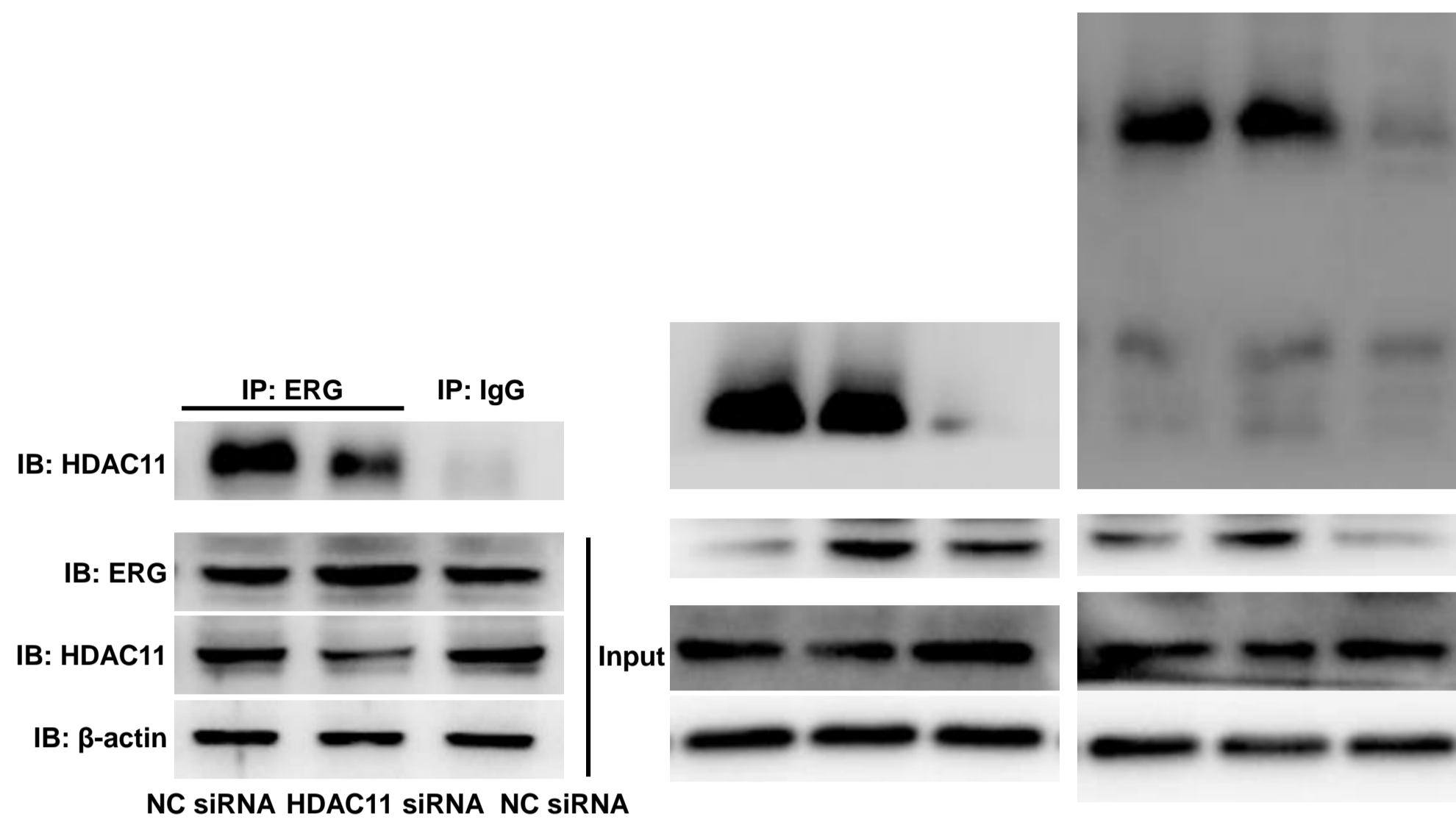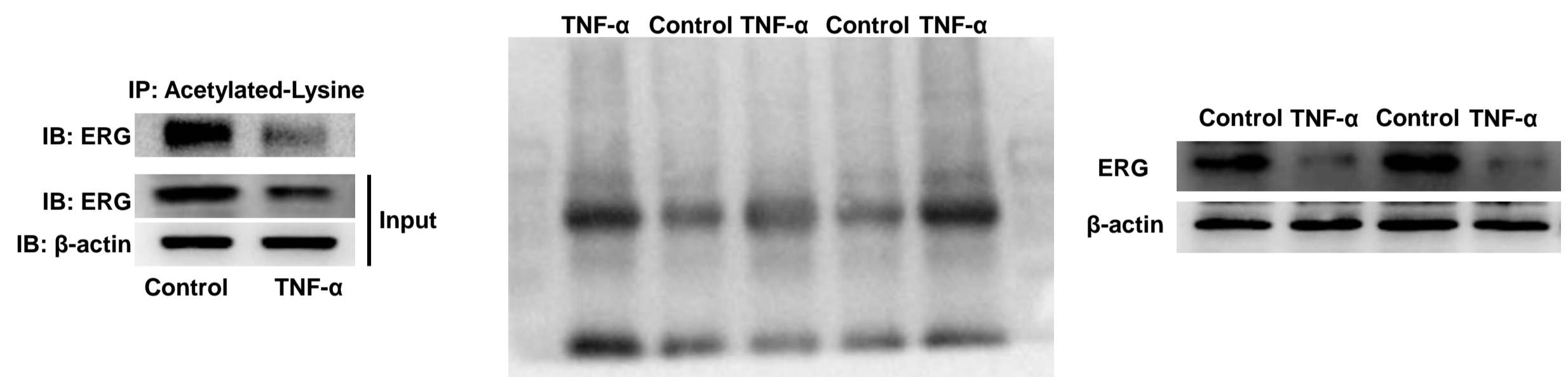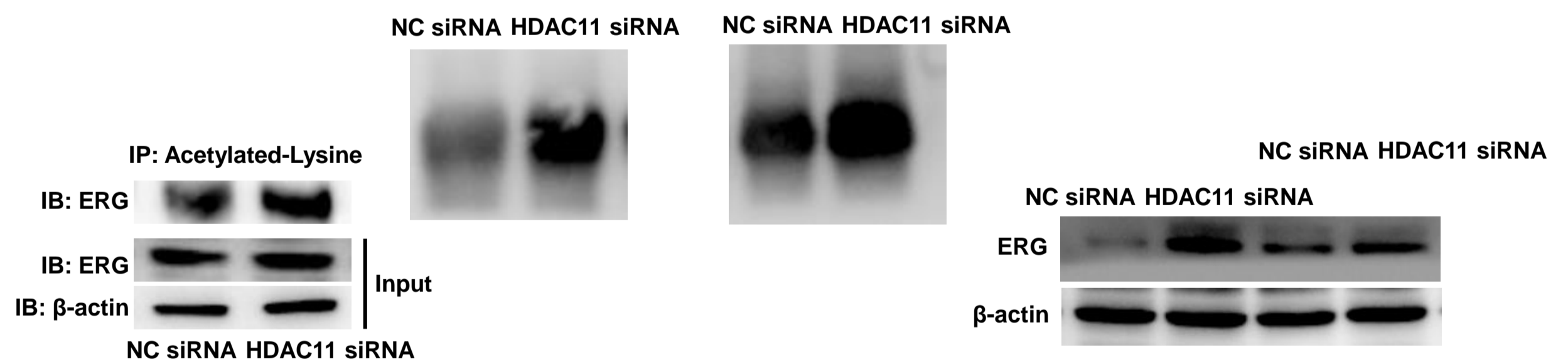

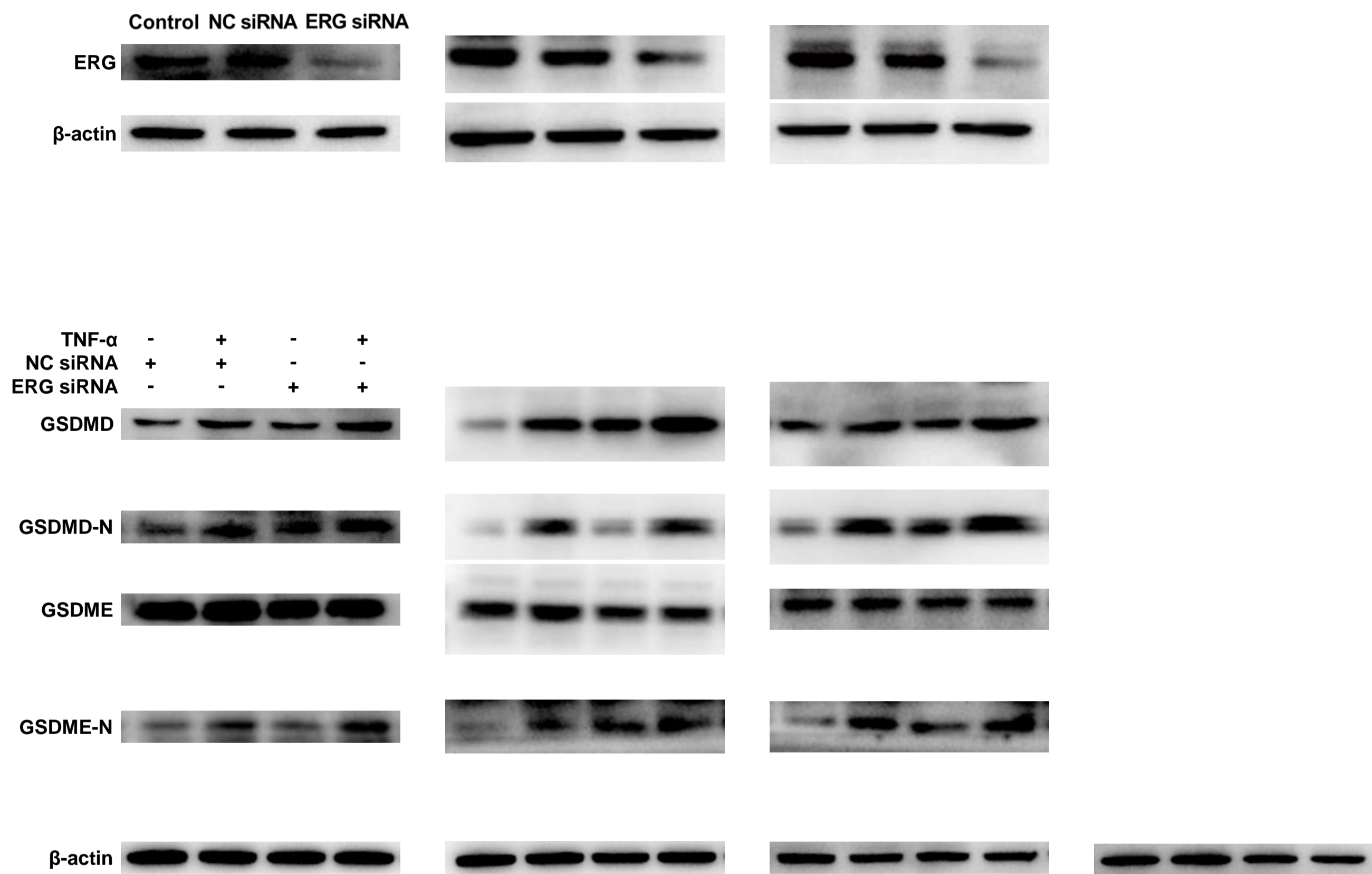

Supplement: Supplementary file 5 — Original Data File [file 41420_2022_906_MOESM5_ESM.pdf]
